# The Performance of Wearable Device–Based Artificial Intelligence in Detecting Depression: Systematic Review and Meta-Analysis

**DOI:** 10.2196/85319

**Published:** 2026-03-10

**Authors:** Jiawen Liu, Junhui Wang, Zhaobin Wu, Mohamad Ibrani Shahrimin Bin Adam Assim

**Affiliations:** 1 Liuzhou Railway Vocational Technical College, 2 Wenyuan Road, Yufeng District, Liuzhou, 545000, China, 60 11 1667 0058; 2 Faculty of Humanities, Management and Science, Universiti Putra Malaysia, Sarawak, Malaysia; 3 School of Mechanical and Automotive Engineering, Guangxi University of Science and Technology, Liuzhou, China; 4 School of Automation, Guangxi University of Science and Technology, Liuzhou, China

**Keywords:** wearable device, artificial intelligence, depression detection, depressive episode prediction, meta-analysis

## Abstract

**Background:**

In recent years, advances in wearable sensor technology and artificial intelligence (AI) have provided new possibilities for detecting and monitoring depression.

**Objective:**

This study systematically reviewed and meta-analyzed the diagnostic and predictive performance of wearable device–based AI models for detecting depression and predicting depressive episodes and explored factors influencing outcomes.

**Methods:**

Following PRISMA-DTA (Preferred Reporting Items for a Systematic Review and Meta-Analysis of Diagnostic Test Accuracy) guidelines, the PubMed, Embase, Web of Science, and PsycINFO databases were searched from inception to May 27, 2025. Eligible studies used AI algorithms on wearable device data for depression detection or episode prediction. Sensitivity, specificity, diagnostic odds ratio, and area under the curve (AUC) were pooled using a bivariate random effects model. Risk of bias was assessed using Prediction Model Risk of Bias Assessment Tool plus artificial intelligence (PROBAST+ AI), and certainty of evidence was assessed using the Grading of Recommendations Assessment, Development, and Evaluation (GRADE) tool.

**Results:**

We included 16 studies (32 datasets) with 1189 patients and 13,593 samples. For depression detection, pooled sensitivity and specificity were 0.89 (95% CI 0.83‐0.93) and 0.93 (95% CI 0.87‐0.96), with a diagnostic odds ratio of 110.47 (95% CI 33.33‐366.17) and AUC of 0.96 (95% CI 0.94‐0.98). Random forest models showed the best performance (sensitivity=0.89, specificity=0.91, AUC=0.97). Subgroup analyses indicated that study design, AI method, reference standard, and input type significantly affected diagnostic accuracy (*P*<.05). For depressive episode prediction (3 datasets), pooled sensitivity was 0.86 (95% CI 0.80‐0.91), and pooled specificity was 0.65 (95% CI 0.59‐0.71). The overall risk of bias was low to moderate, with no evidence of publication bias.

**Conclusions:**

Wearable device–based AI models achieved high accuracy for detecting depression and moderate utility in predicting episodes. However, heterogeneity, reliance on retrospective and public datasets, and lack of standardized methods limited generalizability.

## Introduction

Depression is a highly prevalent psychiatric disorder. According to World Health Organization (WHO) statistics, there are more than 350 million people with depression worldwide, and it is predicted that, by 2030, depression will become the leading cause of the global disease burden [1]. Patients with depression often experience persistent low mood, anhedonia, sleep disturbances, and cognitive impairment accompanied by a significantly increased risk of self-harm and suicide [2]. Depression not only has a significant impact at the individual level but also imposes a heavy economic burden on health care systems and society as a whole [3].

Traditionally, the diagnosis of depression relies on standardized clinical criteria and rating scales. The *Diagnostic and Statistical Manual of Mental Disorders*, *Fourth Edition* (DSM-IV) and *Fifth Edition* (DSM-V) provide operationalized criteria. These still depend on subjective symptom assessment and patient self-report, making them susceptible to reporting bias and interrater variability [4]. The Hamilton Depression Rating Scale (HDRS) and Montgomery-Åsberg Depression Rating Scale (MADRS) are commonly used to quantify disease severity. However, their accuracy depends on the clinician’s expertise and may vary due to different interpretations of symptom severity [5]. The Patient Health Questionnaire-9 (PHQ-9) is widely used in clinical and research settings due to its ease of administration; however, it remains limited by recall bias and the patient’s willingness or ability to express psychological distress accurately [6]. Although these scale and interview-based diagnostic methods are well-established, they lack objective biomarkers. They are limited in providing real-time and ecologically valid assessments, especially as symptom presentation may fluctuate over short periods.

In recent years, advances in wearable sensor technology and artificial intelligence (AI) have provided new possibilities for detecting and monitoring depression [7]. Wearable devices, such as wristbands and smartwatches, can collect longitudinal, multimodal physiological and behavioral data (eg, heart rate variability, sleep patterns, skin temperature, geolocation) [8,9]. This provides a more objective and continuous approach to assessing depressive symptoms. AI methods based on these data sources have demonstrated promising accuracy for depression classification and severity prediction, with studies reporting identification accuracies ranging from approximately 76% to >90% depending on the sample size, data type, and analytical strategy [10,11]. However, significant differences exist in the diagnostic performance reported across studies, largely due to variations in algorithms, wearable devices, and study populations [12,13]. Prior reviews, such as that by Abd-Alrazaq et al [14], have examined digital or sensor-based approaches for mental health detection, but these have mainly focused on feasibility or cross-sectional screening rather than on the predictive capacity of wearable device–based AI for future depressive episodes [15-17]. As systematic evaluations centered specifically on wearable-derived physiological and behavioral data remain limited, our review adds value by assessing the ability of wearable AI models to forecast depressive episodes, incorporating additional summary metrics such as the area under the curve (AUC) and diagnostic odds ratio (DOR) for a more comprehensive evaluation, and conducting subgroup analyses by algorithm type to clarify how methodological factors influence diagnostic performance.

We aimed to conduct a systematic review and meta-analysis to comprehensively assess the diagnostic performance of wearable device–based AI in depression detection and depressive episode prediction. Furthermore, we sought to evaluate how this performance is influenced by key methodological variables through subgroup analyses, focusing on factors such as study design, reference standard, AI algorithm type, and data source.

## Methods

The meta-analysis rigorously adhered to the PRISMA-DTA (Preferred Reporting Items for Systematic Reviews and Meta-Analyses of Diagnostic Test Accuracy) reporting guidelines [18]. The completed checklist is available as Checklist 1. Prior to study initiation, the research protocol was registered in the PROSPERO registry (registration ID: CRD420251070778).

### Ethical Considerations

Because this is a systematic review and meta-analysis, ethics approval and consent to participate were not applicable. The manuscript does not include any identifiable participant image nor other personal or clinical details.

### Search Strategy

A comprehensive literature search was conducted in 4 electronic databases: PubMed, Embase, Web of Science, and PsycINFO. The search was finalized on May 27, 2025. The search strategy incorporated 3 key groups of terms: (1) AI-related terms (eg, “artificial intelligence,” “machine learning,” “deep learning”), (2) disease-related terms (eg, “mood disorders,” “depression,” “psychological stress”), and (3) wearable device–related terms (eg, “wearable electronic devices,” “smart watch,” “accelerometer”). Both free-text terms and MeSH terms were used in combination. No restrictions were applied regarding language or publication year during the search. Detailed search strategies for each database are provided in Table S1 in Multimedia Appendix 1. In addition to systematic electronic searches, we performed backward and forward reference list checking of all included studies to find relevant publications in similar meta-analyses [14].

### Inclusion and Exclusion Criteria

The PITROS (participants, index text, target conditions, reference standard, outcomes, and settings) framework was developed for the inclusion criteria. The detail is shown in Table 1. Studies were excluded if (1) the title or abstract was not relevant; (2) the publication type was a review, preprint, meta-analysis, conference abstract, or letter to the editor; (3) the study was not published in English; (4) AI was applied solely to predict treatment or intervention effects for depression, rather than detection or diagnosis; (5) data collection was not performed using wearable devices, including cases where only traditional medical equipment, handheld devices (such as smartphones), or implantable devices were used; or (6) data were collected exclusively via questionnaires or interviews without wearable device input. The screening process was conducted independently by two reviewers (JL and JW), with initial selection based on titles and abstracts, followed by a full-text assessment according to the inclusion and exclusion criteria. Duplicate references were identified and removed using EndNote and manual verification. Discrepancies were resolved through discussion and, if unresolved, by consulting a third reviewer (ZW).

**Table 1.** Summary of the inclusion criteria using the PITROS framework.

Criteria	Details
Participants (P)	Individuals with a clinical diagnosis of depression as well as healthy controls
Index test (I)	Use of noninvasive wearable devices (eg, smartwatches) to collect physiological data for the development and evaluation of artificial intelligence (AI) algorithms aimed at detecting depression or predicting a depressive episode
Target conditions (T)	Positive group: individuals who met the standardized criteria for depression or those experiencing a depressive episode; negative group: healthy individuals or those not experiencing an episode
Reference standard (R)	Validated diagnostic scales or criteria such as the Montgomery-Åsberg Depression Rating Scale (MADRS); Patient Health Questionnaire-9 (PHQ-9); *Diagnostic and Statistical Manual of Mental Disorders, Fourth Edition* (DSM-IV); *Diagnostic and Statistical Manual of Mental Disorders, Fifth Edition* (DSM-V); and Hamilton Depression Rating Scale (HDRS) used to verify wearable device–based AI algorithm performance
Outcomes (O)	Sensitivity, specificity, diagnostic odds ratio (DOR), and area under the curve (AUC)
Settings (S)	Retrospective or prospective studies conducted in contexts such as public databases or local hospitals

### Quality Assessment

The quality assessment of the included studies was performed using the latest Prediction Model Risk of Bias Assessment Tool plus artificial intelligence (PROBAST+ AI) tool, which has replaced the previous PROBAST 2019 version [19]. This comprehensive instrument is structured into 2 phases—model development and model evaluation—with each phase comprising 7 domains. These domains address key aspects, including participants and data sources, predictors, outcome assessment, and analysis. Each domain is rated as a low (L), high (H), or unclear (U) risk of bias based on clearly defined signaling questions. The signaling questions are categorized as “Yes,” “Probably Yes,” “Probably No,” “No,” “No Information,” and, where applicable, “Not Applicable.” A domain is considered at low risk of bias if all signaling questions are answered with “Yes” or “Probably Yes.” Conversely, the presence of any “No” or “Probably No” responses in a domain indicates a potential high risk of bias. If neither of these are present but “No Information” is shown, the risk of bias is classified as unclear. Detailed signaling questions and summary tables are provided in Multimedia Appendix 1 (Table S2 and Table S3).

 To improve the accuracy during the quality assessment process, two reviewers (JL and JW) independently evaluated the risk of bias for each included study using the PROBAST+ AI tool. In cases where discrepancies arose, consensus was achieved through discussion and critical analysis.

### Certainty of Evidence

The Grading of Recommendations, Assessment, Development, and Evaluation (GRADE) framework was used to assess the certainty of evidence for sensitivity, specificity, and DOR in detecting depression [20]. The evaluation focused on 5 key domains: risk of bias, inconsistency, indirectness, imprecision, and publication bias. If sufficient rationale was identified in any of these domains, the certainty of evidence was downgraded accordingly. Detailed assessment criteria and procedures are provided in Table S4 in Multimedia Appendix 1.

### Data Extraction

Data extraction was conducted independently by 2 reviewers (JL and JW) who assessed the full-text articles to determine their potential eligibility for inclusion. In instances of disagreement, a third reviewer (ZW) acted as an arbitrator to achieve consensus. Extracted data encompassed study characteristics, patient characteristics, and technical parameters, including first author, year of publication, country, study design, target condition, reference standard, age, number of female participants, total participants, the positive sample size, and total sample size. Additionally, information specific to AI technical information was collected, such as the name of wearable devices, placement of wearable devices, data input, dataset source, AI method, AI algorithm, validation approach, and diagnostic performance measures.

True positive (TP) was defined as cases in which the AI model, based on wearable device data, identified depression or a depressive episode, and this was confirmed using the reference standard (MADRS, HDRS, PHQ-9, DSM-IV, or DSM-V). True negative (TN) was defined as nondepressed cases or a nondepressive episode as determined using both the AI model and the reference standard. False positive (FP) referred to cases where the AI model identified depression or a depressive episode, but this was not confirmed using the reference standard. Conversely, false negative (FN) referred to cases where the AI model failed to identify depression or depressive episodes that were confirmed using the reference standard. For eligible studies included in the systematic review but lacking sufficient data for meta-analysis, the corresponding authors were contacted via email to obtain the necessary information. If diagnostic contingency tables were not directly available in the publications, TP, FP, FN, and TN values were primarily back-calculated using reported sensitivity, specificity, the positive sample sizes identified using the reference standard, and the total sample size.

### Outcome Measures

The core outcome parameters analyzed in this systematic review consisted of sensitivity, specificity, DOR, and AUC values extracted from internal validation populations. Sensitivity metrics, also designated as recall or TP rates, quantified the AI model’s capability to properly identify confirmed depression cases or a depressive episode, calculated as TP/(TP+ FN). Specificity, representing TN rates, reflected the algorithm’s accuracy for recognizing nondepressed participants or a nondepressive episode determined via TN/(TN+ FP). AUC represents the area under the receiver operating characteristic curve and serves as a comprehensive measure of the model’s ability to distinguish between positive (depressed or depressive episode) and negative (nondepressed or nondepressive episode) cases. The DOR, an integrative measure of diagnostic performance, combines both sensitivity and specificity, expressing the odds of a positive test result among patients with depression relative to those without depression [21]. For studies evaluating multiple types of AI algorithms, all relevant results were extracted to facilitate direct comparison across different algorithmic methods.

### Statistical Analysis

A bivariate random effects model was used to meta-analyze and assess the diagnostic performance [22]. Forest plots were used to visually present the pooled sensitivity, specificity, and DOR for internal validation datasets, while a summary receiver operating characteristic curve was constructed to display the 95% confidence and prediction regions for the overall estimates [23]. The prediction region indicates the likely range of sensitivity and specificity for future studies, and the pooled AUC was also computed. Heterogeneity across studies was assessed using the Higgins *I*² statistic, with values of 25%, 50%, and 75% denoting low, moderate, and high heterogeneity, respectively [24]. For internal validation datasets with substantial heterogeneity (*I*²>50%), we used bivariate boxplots to identify outliers beyond the 95% CI and to explore potential sources of heterogeneity. In addition, according to the predefined protocol, subgroup analyses were conducted using *z* tests to evaluate the impact of potential variables on the results. Variables included study design (retrospective vs prospective), reference standard (MADRS vs other reference standards), AI method (machine learning vs deep learning), type of input data (only activity data vs others), and data source (open vs closed).

Radar plots were generated to illustrate the distribution of algorithms among the included studies, and bubble plots were used to visualize the trends in the DOR of AI models over time. The Fagan nomogram was applied to evaluate the clinical impact of the AI models [25]. Assessment of publication bias was performed using the Deeks funnel plot asymmetry test, involving a regression of the adequate sample size against the log DOR. All statistical analyses were conducted using the “midas” and “metadta” packages in Stata version 15.1 and R version 4.3.1. A *P* value <.05 was considered statistically significant.

## Results

### Study Selection

**Figure 1.**
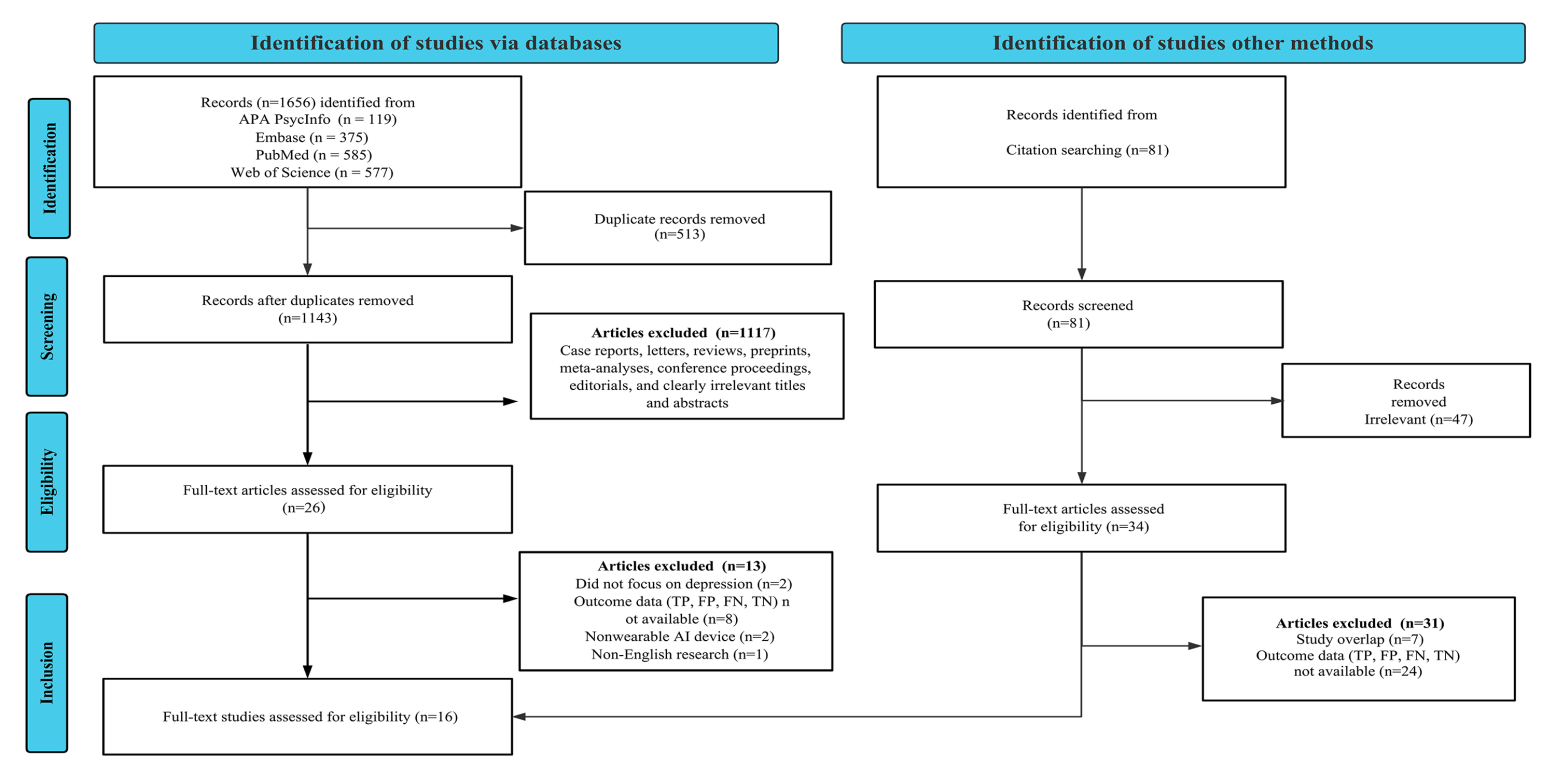
PRISMA (Preferred Reporting Items for Systematic Reviews and Meta-Analyses) flow diagram illustrating the study selection process. AI: artificial intelligence, FN: false negative, FP: false positive, TN: true negative, TP: true positive.

A total of 1656 potentially relevant articles were identified through the searches of the 4 primary databases. After removing 513 duplicates, 1143 unique records remained for preliminary screening. During this phase, 1114 records were excluded due to apparent irrelevance, as determined by their titles and abstracts, or because they did not meet the required publication types. As a result, 60 articles proceeded to the full-text review. Following a detailed assessment, exclusions included the following: 32 studies for insufficient or incomplete diagnostic data (lack of TP, FP, FN, or TN information), 7 for overlapping populations, 2 for not focusing on depression, 2 for not using wearable devices for data collection, and 1 for being published in a language other than English. In addition, we identified 3 records through nondatabase sources. Ultimately, 16 studies fulfilled all inclusion criteria and were included in the meta-analysis [10-13,16,17,26-35]. The study selection process adhered to the PRISMA guidelines, as detailed in Figure 1.

### Study Description and Quality Assessment

A total of 16 eligible studies were included, encompassing 32 internal validation datasets with an aggregate of 1189 patients and 13,593 samples. Data derived from the Depresjon dataset were used in 11 studies [10,11,13,17,26-30,32,35]. The included studies were published between 2019 and 2023. Geographically, one-half (8/16, 50%) were conducted in the Americas (Mexico: n=6; United States, n=2) [10,11,17,27-29,32,35], while the remainder were carried out in Asia (Japan, n=2; China, n=1; Korea, n=1; India, n=1; Saudi Arabia, n=1) [12,13,16,31,33,34] and Europe (Poland, n=1; Norway, n=1) [26,30]. The study designs comprised 10 retrospective [10,11,13,26-30,32,35] and 6 prospective [12,16,17,31,33,34] studies. Regarding reference standards, 10 studies used the MADRS [10,11,13,26-30,32,35], 2 studies used the PHQ-9 [16,17], 1 study used the DSM-IV [34], 1 study used the DSM-V [33], 1 study used the HDRS [12], and 1 study used both the DSM-V and the PHQ-9 [31]. Depression diagnosis was evaluated in 14 studies [10-13,26-35], whereas 2 focused on the identification of depressive episodes [16,17].

Data collection was primarily performed using wrist-worn wearable devices (15 studies) [10-13,16,17,26-33,35], while head-worn devices were used in 1 study [34]. In 10 studies, the data input consisted solely of activity data [10,11,13,26-30,32,35], A combination of activity and sleep data was used in 1 study [33], 1 study relied on electrocardiogram data [34] , and 4 studies incorporated more than 3 types of parameters such as activity data, sleep data, and heart rate for model training [12,16,17,31]. The majority of studies (13/16, 81%) applied machine learning methods [10-13,16,17,26,28,29,31-33,35], while 2 studies used deep learning methods and 1 study used both machine learning and deep learning methods [30]. Data sources were classified as open in 11 studies [10,11,13,26-30,32,34,35] and closed in 5 studies [12,16,17,31,33]. Summaries of the study, patient, and technical characteristics are provided in Table 2 and Multimedia Appendix 1 (Table S5 and Table S6). Among AI algorithms, random forest (RF) was the most frequently implemented (10/32, 31%), with a detailed distribution of the algorithm shown in Figure S1 in Multimedia Appendix 1.

**Table 2.** Study and patient characteristics of the included studies.

Author	Year	Country	Study design	Target condition	Reference standard	Age (years)	Gender (female), n (%)	Total participants, n	Positive sample size, n	Total sample size, n
Adamczyk et al [26]	2021	Poland	Retrospective	Depression vs healthy	MADRS^a^	40.1^b^	30 (55)	55	23	55
Bai et al [16]	2021	China	Prospective	Depressive episode	PHQ-9^c^	18‐60^d^	NR^e^	261	201	201
Espino-Salinas et al [27]	2022	Mexico	Retrospective	Depression vs healthy	MADRS	40.1^b^	30 (55)	55	36	116
Galván-Tejada et al [28]	2019	Mexico	Retrospective	Depression vs healthy	MADRS	40.1^b^	30 (55)	55	962	2483
Jacobson et al [29]	2019	United States	Retrospective	Depression vs healthy	MADRS	40.1^b^	30 (55)	55	23	55
Jakobsen et al [30]	2020	Norway	Retrospective	Depression vs healthy	MADRS	40.1^b^	30 (55)	55	23	55
Mullick et al [17]	2022	United States	Prospective	Depressive episode	PHQ-9	15.5 (12‐18)^f^	41 (75)	55	355	470
Narziev et al [31]	2020	Korea	Prospective	Depression vs healthy	DSM‐5^g^, PHQ-9	NR	NR	20	430	600
Pacheco-González et al [10]	2019	Mexico	Retrospective	Depression vs healthy	MADRS	40.1^b^	30 (55)	55	23	55
Rodríguez-Ruiz et al [32]	2020	Mexico	Retrospective	Depression vs healthy	MADRS	40.1^b^	30 (55)	55	1229	3574
Rodríguez-Ruiz et al [11]	2022	Mexico	Retrospective	Depression vs healthy	MADRS	40.1^b^	53 (49)	109	439	1293
Sato et al [33]	2023	Japan	Prospective	Depression vs healthy	DSM-5	35.6 (11.3)^h^	36 (52)	69	40	69
Sharma et al [34]	2023	India	Prospective	Depression vs healthy	DSM-4^i^	39.3 (12‐77)^f^	24 (38)	64	34	64
Tazawa et al [12]	2020	Japan	Prospective	Depression vs healthy	HDRS^j^	60.2^b^	40 (47)	86	112	236
Zanella-Calzada et al [35]	2019	Mexico	Retrospective	Depression vs healthy	MADRS	40.1^b^	30 (55)	55	1654	4125
Zakariah and Alotaibi [13]	2023	Saudi Arabia	Retrospective	Depression vs healthy	MADRS	20‐69^d^	30 (55)	55	46	142

a MADRS: Montgomery-Åsberg Depression Rating Scale.

b Mean.

c PHQ-9: Patient Health Questionnaire-9.

d Range.

e NR: not reported.

f Median (range).

g DSM-5: *Diagnostic and Statistical Manual of Mental Disorders, Fifth Edition.*

h Mean (SD).

i DSM-4: *Diagnostic and Statistical Manual of Mental Disorders, Fourth Edition.*

j HDRS: Hamilton Depression Rating Scale.

**Figure 2.**
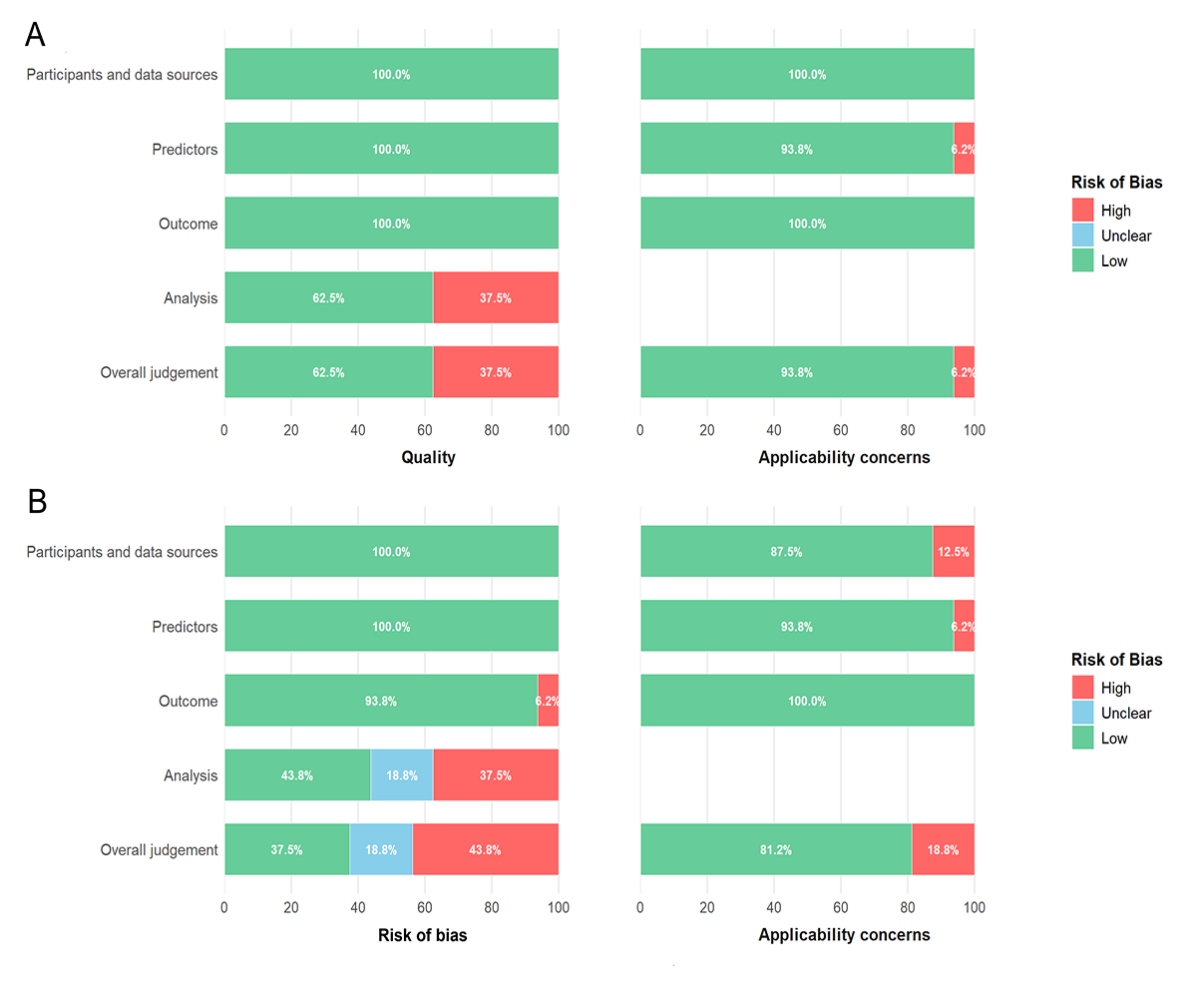
PROBAST+ AI (Prediction Model Risk of Bias Assessment Tool plus artificial intelligence) quality assessment, including risk of bias (high, low, or unclear) of the included studies: (A) model development and (B) model validation.

The risk of bias for the included studies was assessed using the PROBAST+ AI quality assessment tool, with results summarized in Figure 2 and Multimedia Appendix 1 (Table S3 and Table S4). For model development, the overall quality judgment rated 37.5% (6/16) of studies as high risk and the remaining 62% (10/16) as low risk. In terms of applicability concerns, 6% (1/16) of studies were rated as high risk, while 94% (15/16) were rated as low risk. For model validation, the overall risk of bias judgment classified 44% (7/16) of studies as high risk, 19% (3/16) as unclear risk, and 38% (6/16) as low risk. Regarding applicability concerns for model validation, 19% (3/16) of the studies were considered high risk, and 81% (13/16) were rated as low risk. Overall, high-risk items were relatively infrequent, with most studies judged as low risk, indicating that the general methodological quality of the included studies is acceptable. The certainty of evidence, as evaluated using the GRADE framework, ranged from low to high across outcomes, suggesting that the certainty of the evidence was generally moderate (Table S4 in Multimedia Appendix 1).

### Diagnostic Performance of Individual Studies

In the study by Rodríguez-Ruiz et al [32], the detection of depression using an RF model trained on activity data achieved the highest sensitivity (0.99) among all included studies. Their study also reported the highest specificity (0.99) for detecting depression. In addition, for predicting depressive episodes, the study by Bai et al [16] achieved the highest sensitivity (0.90) using a k nearest neighbor (KNN) model trained on activity data, heart rate data, location information, sleep data, smartphone use data, and social interaction data. This study also reported the highest specificity (0.66) for depressive episode prediction using an RF model trained on the same multimodal data sources.

### Diagnostic Performance of Different AI Algorithms for Depression in the Internal Validation Set

Within the internal validation cohorts, the DOR for depression detection using wearable device–based AI algorithms demonstrated a progressive increase from 2019 to 2023, with the highest DOR observed for the Uniform Manifold Approximation and Projection and neural network (UMAP&NN) algorithm and the lowest for the naïve Bayes algorithm (Figure S2 in Multimedia Appendix 1). Subgroup analysis according to algorithm type revealed that the RF algorithm was used in 9 datasets and exhibited robust and promising performance: Sensitivity was 0.89 (95% CI 0.81‐0.94), specificity was 0.91 (95% CI 0.80‐0.96), and the AUC was 0.97 (95% CI 0.95‐0.98; Table S7 in Multimedia Appendix 1).

### Diagnostic Performance of Wearable Device–Based AI Models for Depression in the Internal Validation Set

**Figure 3.**
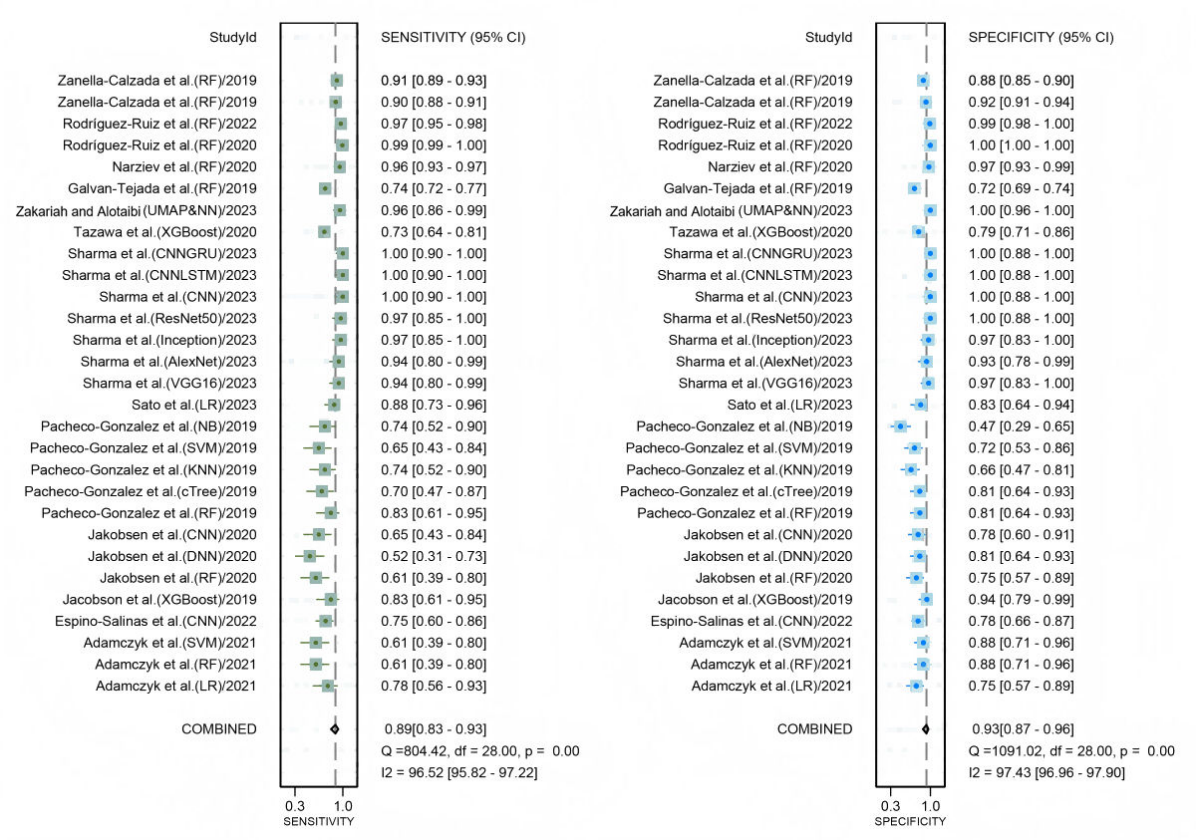
Forest plot of sensitivity and specificity for depression detection using artificial intelligence (AI) in wearable devices, with each row representing an individual study evaluating the performance of different AI algorithms for detecting depression [10-13,26-30,32-35]. Boxes: point estimates; horizontal lines: 95% CIs; diamond at the bottom: pooled estimate. CNN: convolutional neural network; DNN: deep neural network; KNN: k nearest neighbor; LR: logistic regression; LSTM: long short-term memory; NB: naïve Bayes; NN: neural network; RF: random forest; SVM: support vector machine; UMAP: Uniform Manifold Approximation and Projection.

A total of 29 datasets from 14 studies were included in the pooled analysis of diagnostic performance. The combined sensitivity of AI models for depression detection was 0.89 (95% CI 0.83‐0.93; moderate certainty), and the specificity was 0.93 (95% CI 0.87‐0.96; moderate certainty). The pooled DOR was 110.47 (95% CI 33.33‐366.17; low certainty), as illustrated in Figures3  4. Additionally, the AUC for the models was 0.96 (95% CI 0.94‐0.98; Figure 5). Using a pretest probability of 20%, the Fagan nomogram demonstrated a positive likelihood ratio of 76% and a negative likelihood ratio of 3% (Figure S3 in Multimedia Appendix 1). No evidence of publication bias was detected according to the Deeks funnel plot asymmetry test (*P*=.23; Figure S4 in Multimedia Appendix 1).

**Figure 4.**
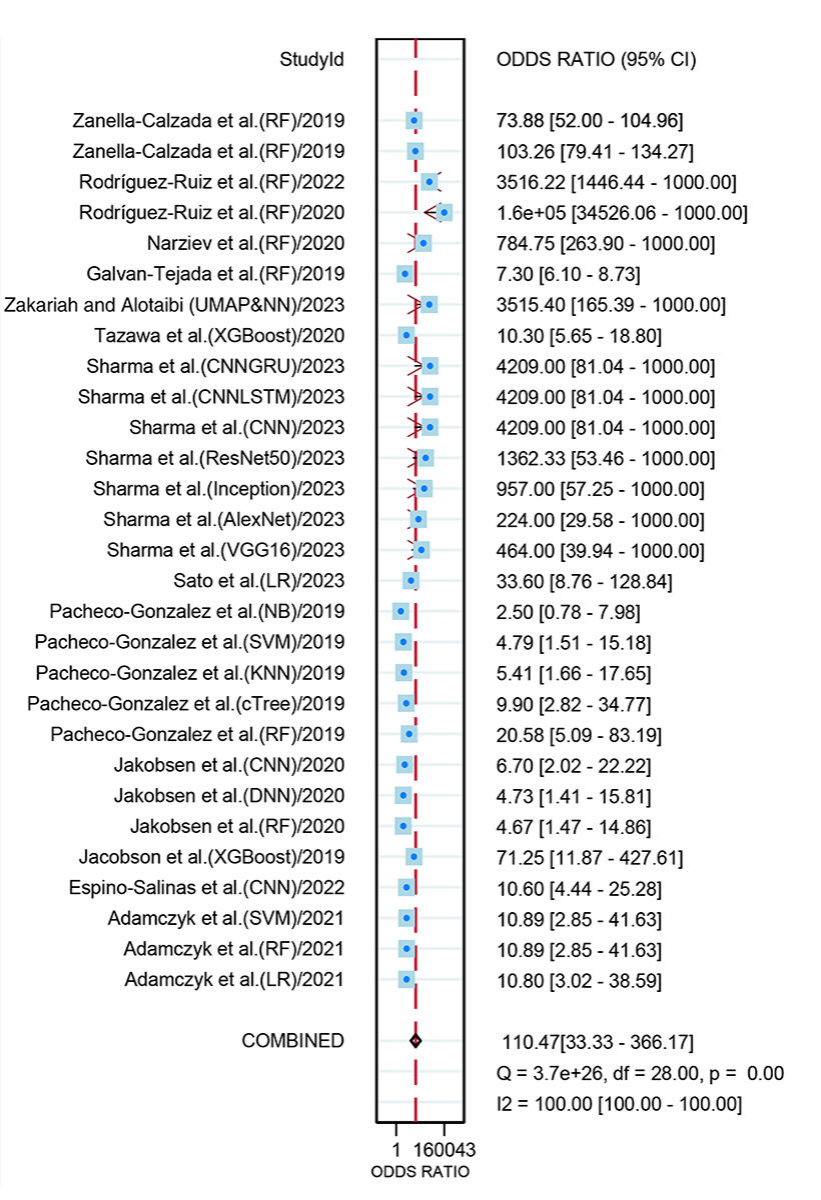
Forest plot of diagnostic performance of wearable device–based artificial intelligence (AI) models for depression in the internal validation set [10-13,26-35]. CNN: convolutional neural network; DNN: deep neural network; KNN: k nearest neighbor; LR: logistic regression; LSTM: long short-term memory; NB: naïve Bayes; NN: neural network; RF: random forest; SVM: support vector machine; UMAP: Uniform Manifold Approximation and Projection.

**Figure 5.**
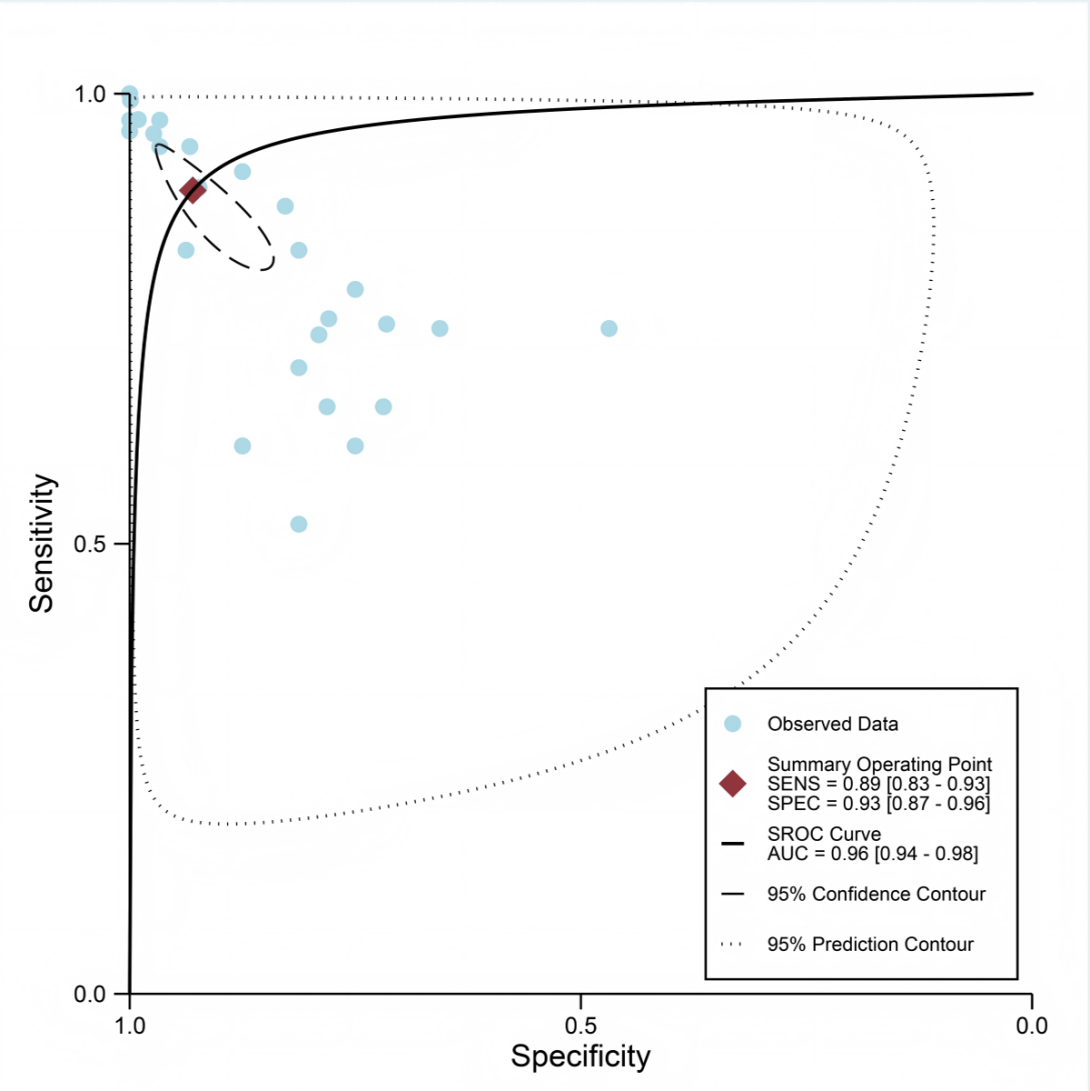
Summary receiver operating characteristic (SROC) curve for depression diagnosis using artificial intelligence (AI) in wearable devices, showing the individual study estimates of sensitivity (SENS) and specificity (SPEC; blue circles) and the summary operating point with pooled sensitivity and specificity and corresponding 95% CIs (red diamond). AUC: area under the curve.

### Bivariate Boxplot and Subgroup Analysis of Wearable Device–Based AI Models in Internal Validation Set

**Figure 6.**
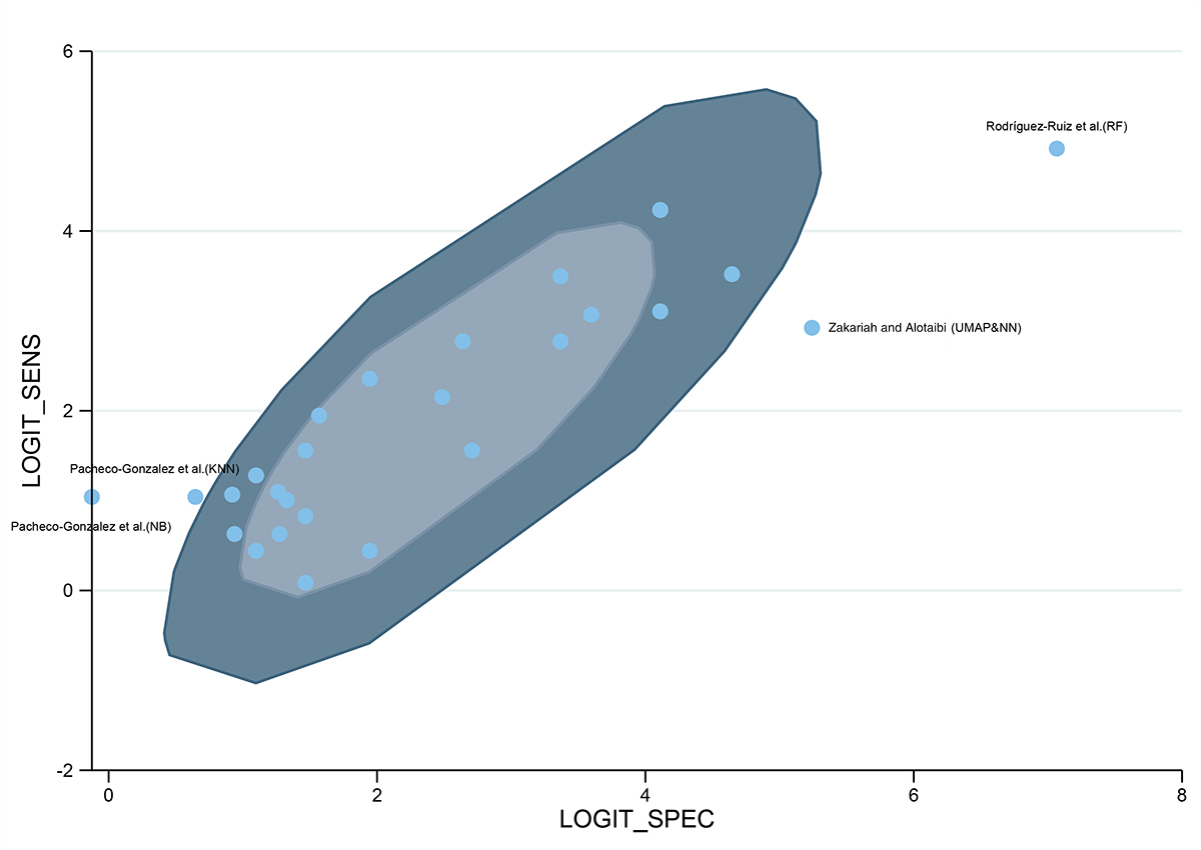
Bivariate boxplot of logit-transformed sensitivity and specificity for wearable device–based artificial intelligence (AI) depression detection [10,13,32]. The inner shaded oval indicates the median distribution of the data points, while the outer shaded oval represents the 95% confidence boundary. KNN: k nearest neighbor; NB: naïve Bayes; NN: neural network; RF: random forest; UMAP: Uniform Manifold Approximation and Projection.

Substantial heterogeneity was observed in both sensitivity (*I*²=96.52%) and specificity (*I*²=97.43%) for depression diagnosis. The bivariate boxplot illustrates that the results from Pacheco-González et al [10] (naïve Bayes and KNN), Zakariah and Alotaibi [13] (UMAP&NN), and Rodríguez-Ruiz et al [32] (RF) fall outside the 95% confidence region, suggesting that these studies may represent potential sources of heterogeneity (Figure 6). Subgroup analyses further demonstrated significant differences in the sensitivity and specificity of AI models according to study design (retrospective vs prospective), reference standard (MADRS vs other reference standards), AI method (machine learning vs deep learning), and data input (only activity data vs other types of data), with all comparisons yielding *P*<.05 (Table S8 in Multimedia Appendix 1).

### Predictive Performance of Wearable Device–Based AI Models for Depressive Episodes in the Internal Validation Set

A total of 3 datasets from 2 studies were included in the pooled analysis of predictive performance for depressive episodes. The wearable device–based AI models demonstrated a sensitivity of 0.86 (95% CI 0.80‐0.91; high certainty) and specificity of 0.65 (95% CI 0.59‐0.71; high certainty), as shown in Figure S5 in Multimedia Appendix 1.

## Discussion

### Principle Findings

Our results demonstrate that wearable device–based AI achieves promising diagnostic performance in depression diagnosis, with sensitivity, specificity, DOR, and AUC of 0.89, 0.93, 110.47, and 0.96, respectively, showing extremely high diagnostic performance. The superior performance of wearable AI systems in depression detection can be attributed to several key mechanisms. First, these devices enable continuous and objective monitoring of multiple physiological and behavioral parameters intrinsically related to depressive symptoms, including motor activity data, sleep data, heart rate data, and disruptions to the circadian rhythm [35,36]. Studies consistently show that, compared with healthy controls, patients with depression exhibit significantly reduced motor activity, altered sleep patterns, and decreased step counts, with these differences being particularly pronounced during specific periods (11 AM to 6 PM) [7]. Second, machine learning algorithms, particularly when combined with dimensionality reduction techniques such as UMAP&NN and ensemble methods like RF and deep neural networks, can identify complex, nonlinear relationships among multiple sensor modalities that may not be apparent in traditional clinical assessments [13]. The integration of features such as skin temperature and sleep duration–related parameters, along with their correlations, has proven particularly valuable in predictive models [37]. Third, the longitudinal nature of wearable data collection enables the detection of subtle changes in behavioral patterns over time, allowing for the early identification of depressive episodes even before their clinical manifestations. This continuous monitoring approach minimizes the biases inherent in traditional self-report measures and provides a more comprehensive assessment of patient status [38].

Our results show that wearable device–based AI has a sensitivity of 0.86 and a specificity of 0.65 for detecting depressive episodes, indicating relatively low specificity. The low specificity of wearable AI systems for detecting depressive episodes may be attributed to several factors. First, in the studies we included, patient populations were patients with major depression who tend to have more complex clinical presentations and comorbidities, which may lead to the overlap of physiological signaling patterns with other psychiatric or physical disorders, thus increasing the rate of FPs [16,17,39]. Additionally, data collected by wearable devices are often affected by user compliance, device wearing habits, and external interferences, which can lead to increased data noise and thus affect the specificity of the model [40]. However, it is important to note that our study on depressive episodes had only 3 datasets and a relatively small sample size, which may affect the stability and reliability of the results. Small sample sizes limit the efficacy of statistical analyses, potentially leading to wider CIs and increased uncertainty in the results. Future prospective studies with larger sample sizes are needed to obtain more robust results.

Interestingly, the results of our subgroup analyses showed that both sensitivity and specificity from prospective studies were significantly higher than those from retrospective studies. The superior diagnostic performance demonstrated by prospective studies in wearable AI depression diagnosis can be attributed to several key mechanisms. First, the prospective study design enables researchers to standardize control over the data collection process, ensuring consistency in wearable device configuration, wearing time, and data quality, whereas retrospective studies often face inconsistent data collection standards. Second, prospective studies can minimize selection bias and recall bias because patients and controls are identified at the beginning of the study, thereby avoiding the risk of sample selection based on known outcomes [12].

Consistent with previous knowledge, our subgroup analysis results showed that both sensitivity and specificity from deep learning algorithms were significantly higher than those from traditional machine learning algorithms. The mechanism by which deep learning algorithms exhibit superior diagnostic performance in wearable AI depression detection can be attributed to their unique architectural advantages and data processing capabilities. First, deep learning models such as deep neural networks and convolutional neural networks possess powerful automatic feature extraction capabilities. This allows them to learn and recognize complex nonlinear patterns directly from raw wearable device data. In contrast, traditional machine learning methods, such as RF, tend to rely on manually engineered statistical features [41]. Second, deep learning models can capture higher-order interactions and temporal dependencies in the data through a multilayer neural network structure, which is crucial for understanding the complex physiological and behavioral patterns of individuals with depression [42]. The results of this study are consistent with those of the study by Jakobsen et al [30], where the sensitivity (0.65) and specificity (0.78) of the convolutional neural network algorithm were similarly shown to be superior to that of the RF algorithm (sensitivity=0.61, specificity=0.75) in their study.

Another interesting finding of our meta-analysis was that AI models trained solely on activity data demonstrated significantly lower sensitivity and specificity than algorithms trained on a combination of multiple data types, such as sleep, heart rate, and activity data. This difference in performance can be attributed to the fact that depression manifests itself through multiple pathophysiological pathways that affect autonomic nervous system function, circadian rhythms, and behavioral patterns [41]. Unimodal approaches may miss critical diagnostic information captured by other sensors, such as heart rate variability, which plays a key role in depression detection as an essential indicator of autonomic dysfunction [43]. The accuracy of the algorithm trained using a combination of multiple datasets (76.67%) was significantly better than that of the AI model trained on activity data (69.24%), as reported by Bai et al [16], further confirming the results of this study.

### Compared With Previous Studies

In 2023, Abd-Alrazaq et al [44] conducted a meta-analysis of wearable device–based AI anxiety tests and reported a sensitivity of 0.79, a specificity of 0.92, and an accuracy of 0.82. Compared with these benchmark results, our study demonstrated that wearable device–based AI depression tests achieved superior diagnostic performance, with sensitivity and specificity of 0.89 and 0.91, respectively. These differences in diagnostic performance may stem from fundamental differences in disease specificity. As noted in the literature, anxiety and depression are related but have different manifestations [44]. Specifically, physiologic indicators of anxiety focus on acute physiologic responses such as elevated heart rate, sweating, and muscle tension, whereas physiologic indicators of depression are more often characterized by chronic changes in sleep patterns, physical activity levels, and mood states [45]. Because of this, chronic physiological changes in depression may be more easily and accurately captured and recognized by existing wearable device technologies than acute physiological responses to anxiety.

Additionally, in 2023, Abd-Alrazaq et al [14] conducted another meta-analysis on the performance of wearable device–based AI for depression detection, which showed a sensitivity of 0.87 and specificity of 0.93. Our results showed that the sensitivity and specificity of wearable device–based AI depression detection were 0.89 and 0.91, respectively, which were generally similar to the results of their study. However, compared with previous studies, our study offers several key innovations. First, it also evaluates the predictive ability for depressive episodes, expanding the scope beyond standard diagnostic assessment. Second, we incorporated additional metrics, such as the AUC and DOR, to provide a more comprehensive evaluation of the diagnostic accuracy of wearable devices. Third, we conducted separate analyses for specific AI algorithms and examined the trends in diagnostic performance over time. Finally, we included more detailed subgroup analyses based on study design, reference standard, AI method, type of input data, and data source, thereby assessing how these factors influence the outcome measures.

### Heterogeneity

Our meta-analysis revealed substantial heterogeneity in the pooled estimates, which is a common and expected finding in meta-analyses of AI-based diagnostic tools. This high degree of heterogeneity reflects the inherent methodological diversity across studies, stemming from variability in datasets, wearable sensor types, data preprocessing pipelines, and AI model architectures. Although our bivariate box-and-whisker plots identified the study by Rodríguez-Ruiz et al [32] as a potential outlier, likely due to its exclusive use of nocturnal data for classification, the overall heterogeneity was multifaceted. Our subgroup analyses further confirmed that study design, AI methodology, input data type, and reference standard significantly influenced diagnostic performance metrics. Therefore, the observed heterogeneity should be interpreted not only as a limitation of the included studies but also as an indicator of the current state of a rapidly evolving and technically diverse research domain.

### Interpreting the Results

When interpreting our results, this study demonstrated that wearable device–based AI exhibits promising performance for diagnosing depression but shows limited effectiveness for predicting depressive episodes. This finding suggests that wearable device–based AI has potential applications in the field of depression diagnosis, and the future deployment of such technologies in daily medical practice may facilitate early identification of and timely intervention in depression. However, it should be clear that these models, at the current level of technology, cannot yet be used as an independent diagnostic tool or basis for clinical decision-making, and the final diagnosis still needs to be comprehensively evaluated by clinical professionals in combination with standardized assessment tools [46]. It is noteworthy that the vast majority of the studies included in the analysis used wrist-worn wearable devices, while only a few studies explored the application of devices on other body parts [34]. This suggests that research on expanding the application sites of wearable devices may be a direction worth exploring in the future. Additionally, the current evidence is based predominantly on internal validation datasets. The general scarcity of rigorous external validation across the literature represents a critical barrier to the clinical translation and real-world reliability of these models, highlighting a key area for future research [45,47].

### Limitations

Several limitations of this meta-analysis should be considered when interpreting the results. First, most of the included studies (10/16, 63%) used a retrospective design, which may have introduced potential selection bias and information bias, as retrospective studies rely on pre-existing data and may have incomplete data or variable quality [48]. Therefore, well-designed prospective studies are needed to validate the findings of this meta-analysis to provide higher-quality evidence. Second, regarding the definition of a gold standard, there were significant differences in the reference standards used across different studies, including variations in depression assessment scales and diagnostic criteria. We attempted to assess the impact of varying gold standards through subgroup analyses, which showed that different subgroup analyses did affect the estimates of sensitivity and specificity, suggesting that the results need to be interpreted with caution and future studies need to be more standardized and consistent in terms of the gold standard qualification [49]. Third, most studies (11/16, 69%) relied on a public dataset (Depresjon datasets) as their primary data sources, which may limit the model’s generalizability and applicability across different populations. In the future, there is a need to collect patient data from more regions and health care organizations to train AI models with more diverse data and validate the external validity of the results [47,50].

### Conclusion

In conclusion, this study demonstrates that wearable device–based AI exhibits promising performance for diagnosing depression but shows limited ability for predicting depressive episodes. Deep learning algorithms and the integration of multimodal data inputs significantly outperformed traditional approaches. However, substantial heterogeneity among studies, along with the predominance of retrospective designs and reliance on public datasets, limits the generalizability of these findings. Future research should focus on large-scale, prospective studies with standardized protocols to enhance clinical applicability and ensure broader external validity.

## Supplementary material

10.2196/85319 Multimedia Appendix 1 Search strategy and additional assessments of the studies.

10.2196/85319 Checklist 1 PRISMA-DTA (Preferred Reporting Items for Systematic Reviews and Meta-Analyses of Diagnostic Test Accuracy) checklist.

## Abbreviations

AI: artificial intelligence
AUC: area under the curve
DOR: diagnostic odds ratio
DSM-IV: *Diagnostic and Statistical Manual of Mental Disorders, Fourth Edition*
DSM-V: *Diagnostic and Statistical Manual of Mental Disorders, Fifth Edition*
FN: false negative
FP: false positive
GRADE: Grading of Recommendations, Assessment, Development, and Evaluation
HDRS: Hamilton Depression Rating Scale
KNN: k nearest neighbor
MADRS: Montgomery-Åsberg Depression Rating Scale
PHQ-9: Patient Health Questionnaire-9
PITROS: participants, index text, target conditions, reference standard, outcomes, and settings
PRISMA-DTA: Preferred Reporting Items for a Systematic Review and Meta-Analysis of Diagnostic Test Accuracy
PROBAST + AI: Prediction Model Risk of Bias Assessment Tool plus artificial intelligence
RF: random forest
TN: true negative
TP: true positive
UMAP&NN: Uniform Manifold Approximation and Projection and neural network
WHO: World Health Organization
