# Supplementary material for: The Performance of Wearable Device–Based Artificial Intelligence in Detecting Depression: Systematic Review and Meta-Analysis
Source: JMIR Ment Health. 2026 Mar 10;13:e85319. doi: 10.2196/85319 (PMC12974932; doi:10.2196/85319)

**Table of Contents**

[Table S1: Search strategy in PubMed, Embase, Web of Science, and APA PsycINFO. 3](#_Toc220261952)

[Table S2: Risk of bias assessment (PROBAST+AI) model development based on seven domains. 6](#_Toc220261953)

[Table S3: Risk of bias assessment (PROBAST+AI) model evaluation based on seven domains. 9](#_Toc220261954)

[Table S4: GRADE scoring assessments in all of the pooled outcomes. 12](#_Toc220261955)

[Table S5: Technical aspects of included studies. 14](#_Toc220261956)

[Table S6: Diagnostic data overview of AI-enabled wearable devices. 16](#_Toc220261957)

[Figure S1: Radar chart of algorithms used in included studies. RF, random forest; NB, Naive Bayes; LR, Logistic Regression; KNN, K-nearest neighbor; CNN, Convolutional Neural network;DNN, Deep Neural Network; AdaBoost Adaptive, boosting; VGG16, Visual geometry group 16-layer; ResNet50, Residual network with 50 Layers; CNN-LSTM, Convolutional neural network - Long short-term memory; CNN-GRU, Convolutional neural network - gated recurrent unit; CNN, Convolutional neural network; Alex Net Alex, krizhevsky’s network; C Tree, Conditional tree; XGBoost, Extreme gradient boosting; UMAP, Uniform manifold approximation and projection; NN, Neural network; SVM, Support vector machine. 1](#_Toc220261958)8

[Figure S2: Bubble chart of changes in diagnostic odds ratio (DOR) over time for different algorithms. The x-axis shows the publication year and the y-axis indicates the DOR. Different colors represent different algorithms. Alex Net Alex, krizhevsky’s network; NB, Naive Bayes; CNN, Convolutional neural network; ResNet50, Residual network with 50 Layers; C Tree, Conditional tree; RF, random forest; DNN, Deep neural network; SVM, Support vector machine; UMAP, Uniform manifold approximation and projection; NN, Neural network; KNN, K-nearest neighbor; VGG16, Visual geometry group 16-layer; LR, Logistic regression; XGBoost, Extreme gradient boosting. 1](#_Toc220261959)9

[Table S7: Meta-analysis based on different AI algorithms. 20](#_Toc220261960)

[Figure S3: Fagan plot of diagnostic performance of wearable device-based AI models for depression in the internal validation set. 21](#_Toc220261961)

[Figure S4: Deeks’ funnel plot assessing publication bias for the diagnostic performance of wearable device-based AI models for depression in the internal validation set. 22](#_Toc220261962)

[Table S8: Subgroup analysis and meta-regression analysis of wearable device-based artificial intelligence performance for detecting depression. 23](#_Toc220261963)

[Figure S5: Forest plot of subgroup analysis for depressive episodes. 24](#_Toc220261964)

Table S1: Search strategy in PubMed, Embase, Web of Science, and APA PsycINFO.

| Database | Search strategy | Filters and Limits | Number of Studies |
| --- | --- | --- | --- |
| PubMed | ("Artificial Intelligence"[MeSH] OR "Artificial Intelligence"[Title/Abstract] OR "Machine Learning"[MeSH] OR "Machine Learning"[Title/Abstract] OR "Deep Learning"[MeSH] OR "Deep Learning"[Title/Abstract] OR "supervised learning"[Title/Abstract] OR "unsupervised learning"[Title/Abstract] OR "reinforcement learning"[Title/Abstract] OR "Decision tree"[Title/Abstract] OR "K-Nearest Neighbor*"[Title/Abstract] OR "Support vector machine*"[Title/Abstract] OR "Recurrent neural network*"[Title/Abstract] OR "convolutional neural network*"[Title/Abstract] OR "Artificial neural network*"[Title/Abstract] OR "Deep Neural Networks"[Title/Abstract] OR "Naïve Bayes"[Title/Abstract] OR "Fuzzy Logic"[Title/Abstract] OR "Random Forest"[Title/Abstract] OR "Long Short-Term Memory Networks"[Title/Abstract] OR "autoencoder"[Title/Abstract] OR "deep belief network"[Title/Abstract] OR "Gradient Boost*"[Title/Abstract] OR "AdaBoost"[Title/Abstract] OR "Multilayer Perceptron"[Title/Abstract] OR "Ensemble learning"[Title/Abstract]) AND ("Mood Disorders"[MeSH] OR "mood disorder*"[Title/Abstract] OR "Depression"[MeSH] OR "depress*"[Title/Abstract] OR "Psychological Distress"[MeSH] OR "distress*"[Title/Abstract] OR "Stress, Psychological"[MeSH] OR "stress*"[Title/Abstract]) AND ("Wearable Electronic Devices"[MeSH] OR "wearable*"[Title/Abstract] OR "smart watch*"[Title/Abstract] OR "smartwatch*"[Title/Abstract] OR "accelerator*"[Title/Abstract] OR "gyroscop*"[Title/Abstract] OR "fitness band*"[Title/Abstract] OR "wristband*"[Title/Abstract] OR "Apple Watch"[Title/Abstract] OR "Fitbit"[Title/Abstract] OR "Samsung Galaxy Watch"[Title/Abstract])z | No restrictions applied regarding date range, language, or publication type. | 585 |
| Embase | ('artificial intelligence'/exp OR 'Artificial Intelligence':ab,ti OR 'Machine Learning'/exp OR 'Machine Learning':ab,ti OR 'Deep Learning'/exp OR 'Deep Learning':ab,ti OR 'supervised learning':ab,ti OR 'unsupervised learning':ab,ti OR 'reinforcement learning':ab,ti OR 'Decision tree':ab,ti OR 'K-Nearest Neighbor*':ab,ti OR 'Support vector machine*':ab,ti OR 'Recurrent neural network*':ab,ti OR 'convolutional neural network*':ab,ti OR 'Artificial neural network*':ab,ti OR 'Deep Neural Networks':ab,ti OR 'Naïve Bayes':ab,ti OR 'Fuzzy Logic':ab,ti OR 'Random Forest':ab,ti OR 'Long Short-Term Memory Networks':ab,ti OR 'autoencoder':ab,ti OR 'deep belief network':ab,ti OR 'Gradient Boost*':ab,ti OR 'AdaBoost':ab,ti OR 'Multilayer Perceptron':ab,ti OR 'Ensemble learning':ab,ti) AND ('depression'/exp OR 'depress*':ab,ti) AND ('wearable computer'/exp OR 'wearable*':ab,ti OR 'smart watch*':ab,ti OR 'smartwatch*':ab,ti OR 'acceleromet*':ab,ti OR 'gyroscop*':ab,ti OR 'fitness band*':ab,ti OR 'wristband*':ab,ti OR 'Apple Watch':ab,ti OR 'Fitbit':ab,ti OR 'Samsung Galaxy Watch':ab,ti) | No restrictions applied regarding date range, language, or publication type. | 375 |
| Web of Science | TS=("Artificial Intelligence" OR "Machine Learning" OR "Deep Learning" OR "supervised learning" OR "unsupervised learning" OR "reinforcement learning" OR "Decision tree" OR "K-Nearest Neighbor*" OR "Support vector machine*" OR "Recurrent neural network*" OR "convolutional neural network*" OR "Artificial neural network*" OR "Deep Neural Networks" OR "Naïve Bayes" OR "Fuzzy Logic" OR "Random Forest" OR "Long Short-Term Memory Networks" OR "autoencoder" OR "deep belief network" OR "Gradient Boost*" OR "AdaBoost" OR "Multilayer Perceptron" OR "Ensemble learning") AND TS=("Depression" OR "depress*") AND TS=("Wearable Electronic Devices" OR "wearable*" OR "smart watch*" OR "smartwatch*" OR "acceleromet*" OR "gyroscop*" OR "fitness band*" OR "wristband*" OR "Apple Watch" OR "Fitbit" OR "Samsung Galaxy Watch") | No restrictions applied regarding date range, language, or publication type. | 577 |
| PsycINFO | ("Artificial Intelligence" OR "Machine Learning" OR "Deep Learning" OR "supervised learning" OR "unsupervised learning" OR "reinforcement learning" OR "decision tree" OR "K-Nearest Neighbor*" OR "support vector machine*" OR "recurrent neural network*" OR "convolutional neural network*" OR "artificial neural network*" OR "deep neural networks" OR "naïve bayes" OR "fuzzy logic" OR "random forest" OR "long short-term memory networks" OR "autoencoder" OR "deep belief network" OR "gradient boost*" OR "adaboost" OR "multilayer perceptron" OR "ensemble learning") AND ("Mood Disorders" OR "mood disorder*" OR "Depression" OR "depress*" OR "Psychological Distress" OR "distress*" OR "Stress" OR "stress*") AND ("Wearable Electronic Devices" OR "wearable*" OR "smart watch*" OR "smartwatch*" OR "acceleromet*" OR "gyroscop*" OR "fitness band*" OR "wristband*" OR "Apple Watch" OR "Fitbit" OR "Samsung Galaxy Watch") | No restrictions applied regarding date range, language, or publication type. | 119 |

Table S2: Risk of bias assessment (PROBAST+AI) model development based on seven domains.

| Author, year | | Quality | | | | Applicability concerns | | | Overall judgement | |
| --- | --- | --- | --- | --- | --- | --- | --- | --- | --- | --- |
|  |  | Participants and data sources ^a^ | Predictors ^b^ | Outcome ^c^ | Analysis ^d^ | Participants and data sources ^e^ | Predictors ^f^ | Outcome ^g^ | Quality ^h^ | Applicability concerns ^i^ |
| Adamczyk et al. | 2021 | L | L | L | H | L | L | L | H | L |
| Espino-Salinas et al. | 2022 | L | L | L | L | L | L | L | L | L |
| Galvan-Tejada et al. | 2019 | L | L | L | L | L | L | L | L | L |
| Jacobson et al. | 2019 | L | L | L | H | L | L | L | H | L |
| Jakobsen et al. | 2020 | L | L | L | H | L | L | L | H | L |
| Narziev et al. | 2020 | L | L | H | L | L | L | L | L | L |
| Pacheco-Gonzalez et al. | 2019 | L | L | L | H | L | L | L | H | L |
| Rodríguez-Ruiz et al. | 2020 | L | L | L | L | L | L | L | L | L |
| Rodríguez-Ruiz et al. | 2022 | L | L | L | L | L | L | L | L | L |
| Sato et al. | 2023 | L | L | L | H | L | L | L | H | L |
| Sharma et al. | 2023 | L | L | L | H | L | H | L | H | H |
| Tazawa et al. | 2020 | L | L | L | L | L | L | L | L | L |
| Zanella-Calzada et al. | 2019 | L | L | L | L | L | L | L | L | L |
| Zakariah et al. | 2023 | L | L | L | L | L | L | L | L | L |
| Bai et al. | 2021 | L | L | L | L | L | L | L | L | L |
| Mullick et al. | 2022 | L | L | L | L | L | L | L | L | L |

**Abbreviation:** PROBAST+AI, Prediction model Risk of Bias Assessment Tool + AI, L low; H high; U unclear.

**Footnote:** Signaling questions are rated as "yes" (Y), "probably yes" (PY), "probably no" (PN), "no" (N), "no information" (NI), and in some cases "not applicable" (NA). All signaling questions are phrased in such a way that "yes" or "probably yes" indicates a low risk of bias. Any signaling questions rated as "no" or "probably no" indicate a potential high risk of bias in that domain. If there are no "no" or "probably no" ratings, but "no information" (NI) is present, the risk of bias in that domain is classified as unclear.

a. Participants and data sources

1.1 Were appropriate data sources used?

1.2 Was an appropriate study design used?

1.3 Did the in- and exclusions of study participants result in a representative dataset?

b. Predictors

2.1 Were predictors defined and assessed in a similar way for all participants?

2.2 Was any pre-processing of predictors similar for all participants?

2.3 Were predictor assessments made without knowledge of outcome data?

2.4 Were the predictors included in the model available at the time the model was intended to be used?

c. Outcome

3.1 Were outcomes defined and assessed appropriately?

3.2 Were outcomes defined and assessed in a similar way for all participants?

3.3 Were outcome assessments made without use or knowledge of predictor data?

3.4 Was the time interval between predictor assessment and outcome assessment appropriate?

d. Analysis

4.1 Was there evidence that the sample size was reasonable?

4.2 Were continuous and categorical predictors handled appropriately?

4.3 Were participants with missing or censored data handled appropriately in the analysis?

4.4 If methods to address class imbalance were used, was the model or the model predictions recalibrated?

4.5 Were methods used to address potential model overfitting?

e. Participants and data sources

Concern that the (data of the) included participants do not match the review question or the assessor’s intended use of the prediction model.

f. Predictors

Concern that the definition, pre-processing, assessment, or timing of assessment of the predictors in the model do not match the review question or the assessor’s intended use.

g. Outcome

Concern that the outcome, its definition, assessment, or timing of assessment do not match the review question or the assessor’s intended use.

h. Quality

Low risk: If all four domains were rated low concern regarding quality.

High risk: If at least one domain was rated high concern regarding quality .

Unclear: If at least one domain was rated unclear concern regarding quality and no domains were rated high concern.

i. Applicability concerns

Low risk: If all three domains were rated low concern for applicability.

High risk: If at least one domain was rated high concern for applicability.

Unclear: If at least one domain was rated unclear concern for applicability and no domains were rated high concern.

Table S3: Risk of bias assessment (PROBAST+AI) model evaluation based on seven domains.

| Author, year | | Risk of bias | | | | Applicability concerns | | | Overall judgement | |
| --- | --- | --- | --- | --- | --- | --- | --- | --- | --- | --- |
|  |  | Participants and data sources ^a^ | Predictors ^b^ | Outcome ^c^ | Analysis ^d^ | Participants and data sources ^e^ | Predictors ^f^ | Outcome ^g^ | Risk of bias ^h^ | Applicability concerns ^i^ |
| Adamczyk et al. | 2021 | L | L | L | H | H | L | L | H | H |
| Espino-Salinas et al. | 2022 | L | L | L | L | L | L | L | L | L |
| Galvan-Tejada et al. | 2019 | L | L | L | U | L | L | L | U | L |
| Jacobson et al. | 2019 | L | L | L | H | L | L | L | H | L |
| Jakobsen et al. | 2020 | L | L | L | H | L | L | L | H | L |
| Narziev et al. | 2020 | L | L | H | L | L | L | L | H | L |
| Pacheco-Gonzalez et al. | 2019 | L | L | L | H | L | L | L | H | L |
| Rodríguez-Ruiz et al. | 2020 | L | L | L | L | L | L | L | L | L |
| Rodríguez-Ruiz et al. | 2022 | L | L | L | L | H | L | L | L | H |
| Sato et al. | 2023 | L | L | L | H | L | L | L | H | L |
| Sharma et al. | 2023 | L | L | L | H | L | H | L | H | H |
| Tazawa et al. | 2020 | L | L | L | L | L | L | L | L | L |
| Zanella-Calzada et al. | 2019 | L | L | L | L | L | L | L | L | L |
| Zakariah et al. | 2023 | L | L | L | L | L | L | L | L | L |
| Bai et al. | 2021 | L | L | L | U | L | L | L | U | L |
| Mullick et al. | 2022 | L | L | L | U | L | L | L | U | L |

**Abbreviation:** PROBAST+AI, Prediction model Risk of Bias Assessment Tool + AI, L low; H high; U unclear.

**Footnote:** Signaling questions are rated as "yes" (Y), "probably yes" (PY), "probably no" (PN), "no" (N), "no information" (NI), and in some cases "not applicable" (NA). All signaling questions are phrased in such a way that "yes" or "probably yes" indicates a low risk of bias. Any signaling questions rated as "no" or "probably no" indicate a potential high risk of bias in that domain. If there are no "no" or "probably no" ratings, but "no information" (NI) is present, the risk of bias in that domain is classified as unclear.

a. Participants and data sources

1.1 Were appropriate data sources used?

1.2 Was an appropriate study design used?

1.3 Did the in- and exclusions of study participants result in a representative dataset?

b. Predictors

2.1 Were predictors defined and assessed in a similar way for all participants?

2.2 Was any pre-processing of predictors similar for all participants?

2.3 Were predictor assessments made without knowledge of outcome data?

2.4 Were the predictors included in the model available at the time the model was intended to be used?

c. Outcome

3.1 Were outcomes defined and assessed appropriately?

3.2 Were outcomes defined and assessed in a similar way for all participants?

3.3 Were outcome assessments made without use or knowledge of predictor data?

3.4 Was the time interval between predictor assessment and outcome assessment appropriate?

d. Analysis

4.1 Was model evaluation based on only apparent performance avoided?

4.2 Was there evidence that the sample size was reasonable?

4.3 Were participants with missing or censored data handled appropriately in the analysis?

4.4 If methods to address class imbalance were used, was the evaluation done in a dataset without imbalance correction?

4.5 If data splitting was done to create training and test datasets, was there evidence that data leakage was avoided?

e. Participants and data sources

Concern that the (data of the) included participants do not match the review question or the assessor’s intended use of the prediction model.

f. Predictors

Concern that the definition, pre-processing, assessment, or timing of assessment of the predictors in the model do not match the review question or the assessor’s intended use.

g. Outcome

Concern that the outcome, its definition, assessment, or timing of assessment do not match the review question or the assessor’s intended use.

h. Risk of bias

Low risk: If all four domains were rated low risk of bias.

High risk: If at least one domain was rated high risk of bias.

Unclear: If at least one domain was rated unclear risk of bias and no domains were rated high risk of bias.

i. Applicability concerns

Low risk: If all three domains were rated low concern for applicability.

High risk: If at least one domain was rated high concern for applicability.

Unclear: If at least one domain was rated unclear concern for applicability and no domains were rated high concern.

Table S4: GRADE scoring assessments in all of the pooled outcomes.

| Data set | Outcome | Risk of Bias ^a^ | Inconsistency ^b^ | Indirectness ^c^ | Imprecision ^d^ | Publication Bias^e^ | Total Downgrade | Final Rating |
| --- | --- | --- | --- | --- | --- | --- | --- | --- |
| Depression Diagnosis | Sensitivity | 1 | 0 | 0 | 0 | 0 | 1 | Moderate |
|  | Specificity | 1 | 0 | 0 | 0 | 0 | 1 | Moderate |
|  | DOR | 1 | 1 | 0 | 0 | 0 | 2 | Low |
| Depressive episodes | Sensitivity | 0 | 0 | 0 | 0 | 0 | 0 | High |
|  | Specificity | 0 | 0 | 0 | 0 | 0 | 0 | High |

a. Risk of Bias

Assessed using tools like PROBAST+AI to evaluate study design and methodology.

Downgrade by 1 level if at least one study has a high risk of bias.

b. Inconsistency

Measured using I² statistics to assess heterogeneity across studies.

Downgrade by 1 level if I² > 50% and the source of heterogeneity cannot be explained.

If the heterogeneity is identified (e.g., through meta-regression), no downgrade is applied.

c. Indirectness

Evaluates whether study populations, interventions, or outcomes differ from the target research question.

Patient Indirectness: Downgrade if the included population significantly deviates from the target population (e.g., specific subgroups).

Outcome Indirectness: Downgrade if inconsistent outcome measures are used, such as estimating sensitivity/specificity indirectly from ROC-based Youden Index.

d. Imprecision

Assessed based on confidence intervals (CIs) and sample size sufficiency.

For sensitivity/specificity: Downgrade if 95% CI width > 0.20 or if CIs cross clinical thresholds (e.g., 0.80).

For DOR: Downgrade if CI range exceeds 1× the lower limit or includes values < 10.

Sample size threshold: Total sample < 500 or positive/negative cases < 100.

e. Publication Bias

Evaluated using Deek’s Funnel Plot or similar tools.

Downgrade by 1 level if the funnel plot shows significant asymmetry or if P-value < 0.05 in Deek’s tests.

Table S5: Technical aspects of included studies.

| Author | Year | Name of WD | Placement of WD | Data input | Data set  source | AI method | AI algorithm | Validation  approach |
| --- | --- | --- | --- | --- | --- | --- | --- | --- |
|  |  |  |  |  |  |  |  |  |
| Adamczyk et al. | 2021 | Act iWatch AW4 | Wrist | Activity data | Open | Machine learning | LR, RF, SVM | Nested |
| Bai et al. | 2021 | Mi Band 2 | Wrist | Activity data,  heart rate data, location,  sleep data, smartphone usage data,  social interaction | Closed | Machine learning | KNN, RF | K-fold |
| Espino-Salinas et al. | 2022 | Act iWatch AW4 | Wrist | Activity data | Open | Deep learning | CNN | Hold-out, K-fold |
| Galvan-Tejada et al. | 2019 | Act iWatch AW4 | Wrist | Activity data | Open | Machine learning | RF | Hold-out, K-fold |
| Jacobson et al. | 2019 | Act iWatch AW4 | Wrist | Activity data | Open | Machine learning | XG Boost | LOOCV |
| Jakobsen et al. | 2020 | Act iWatch AW4 | Wrist | Activity data | Open | Machine learning, Deep learning | RF, DNN, CNN | LOOCV |
| Mullick et al. | 2022 | Fitbit Inspire HR | Wrist | Activity data,  heart rate data, location, sleep data, smartphone usage data, social interaction | Closed | Machine learning | AdaBoost, RF, XG Boost | LOOCV |
| Narziev et al. | 2020 | Gear S3 | Wrist | Activity data, light exposure, food intake, heart rate data, mood status, sleep data, smartphone use data, social interaction | Closed | Machine learning | RF | Hold-out, K-fold |
| Pacheco-Gonzalez et al. | 2019 | Act iWatch AW4 | Wrist | Activity data | Open | Machine learning | RF, cTree, KNN, SVM, Nave Bayes | NR |
| Rodríguez-Ruiz et al. | 2020 | Act iWatch AW4 | Wrist | Activity data | Open | Machine learning | RF | Hold-out |
| Rodríguez-Ruiz et al. | 2022 | Act iWatch AW4 | Wrist | Activity data | Open | Machine learning | RF | K-fold |
| Sato et al. | 2023 | E4 wristband | Wrist | Activity data, sleep data | Closed | Machine learning | LR | 10-fold cross-validation |
| Sharma et al. | 2023 | Dep Cap | Head | EEG | Open | Deep learning | VGG16, Alex Net, Inception, ResNet50, CNN, CNN-LSTM, CNN-GRU | 10-fold cross-validation |
| Tazawa et al. | 2020 | Silmee W20 | Wrist | Activity data,  heart rate data,  skin temperature, sleep data,  UV light exposure | Closed | Machine learning | XG Boost | K-fold |
| Zanella-Calzada et al. | 2019 | Act iWatch AW4 | Wrist | Activity data | Open | Machine learning | RF | Hold-out |
| Zakariah et al. | 2023 | Act iWatch AW4 | Wrist | Activity data | Open | Machine learning | UMAP, NN | LOOCV |

LR Logistic regression; RF Random forest; SVM Support vector machine; CNN Convolutional neural network; DNN Deep neural network; XGBoost Extreme gradient boosting; AdaBoost Adaptive boosting; C Tree Conditional tree; KNN K-nearest neighbor; Alex Net Alex krizhevsky’s network; VGG16 Visual geometry group 16-layer; ResNet50 Residual network with 50 Layers; CNN-LSTM Convolutional neural network - Long short-term memory; CNN-GRU Convolutional neural network - gated recurrent unit; UMAP Uniform manifold approximation and projection; NN Neural network; LOOCV Leave-one-out cross-validation; NR not reported; EEG Electroencephalography.

Table S6: Diagnostic data overview of AI-enabled wearable devices.

| Author | Year | AI algorithms | AI algorithm | | | |
| --- | --- | --- | --- | --- | --- | --- |
|  |  |  | TP | FP | TN | FN |
| Adamczyk et al. | 2021 | LR | 18 | 8 | 24 | 5 |
|  |  | RF | 14 | 4 | 28 | 9 |
|  |  | SVM | 14 | 4 | 28 | 9 |
| Bai et al. | 2021 | KNN | 71 | 43 | 79 | 8 |
|  |  | RF | 71 | 41 | 81 | 8 |
| Espino-Salinas et al. | 2022 | CNN | 36 | 15 | 53 | 12 |
| Galvan-Tejada et al. | 2019 | RF | 962 | 326 | 819 | 331 |
| Jacobson et al. | 2019 | XGBoost | 19 | 2 | 30 | 4 |
| Jakobsen et al. | 2020 | RF | 14 | 8 | 24 | 9 |
|  |  | DNN | 12 | 6 | 26 | 11 |
|  |  | CNN | 15 | 7 | 25 | 8 |
| Mullick et al. | 2022 | AdaBoost & RF & XGBoost | 355 | 14 | 24 | 77 |
| Narziev et al. | 2020 | RF | 430 | 4 | 146 | 20 |
| Pacheco-Gonzalez et al. | 2019 | RF | 19 | 6 | 26 | 4 |
|  |  | cTree | 16 | 6 | 26 | 7 |
|  |  | KNN | 17 | 11 | 21 | 6 |
|  |  | SVM | 15 | 9 | 23 | 8 |
|  |  | NaveBayes | 17 | 17 | 15 | 6 |
| Rodríguez-Ruiz et al. | 2020 | RF | 1229 | 2 | 2344 | 9 |
| Rodríguez-Ruiz et al. | 2022 | RF | 439 | 8 | 833 | 13 |
| Sato et al. | 2023 | LR | 35 | 5 | 24 | 5 |
| Sharma et al. | 2023 | VGG16 | 32 | 1 | 29 | 2 |
|  |  | AlexNet | 32 | 2 | 28 | 2 |
|  |  | Inception | 33 | 1 | 29 | 1 |
|  |  | ResNet50 | 33 | 0 | 30 | 1 |
|  |  | CNN | 34 | 0 | 30 | 0 |
|  |  | CNN-LSTM | 34 | 0 | 30 | 0 |
|  |  | CNN-GRU | 34 | 0 | 30 | 0 |
| Tazawa et al. | 2020 | XG Boost | 82 | 26 | 98 | 30 |
| Zanella-Calzada et al. | 2019 | RF | 1135 | 114 | 1369 | 132 |
|  |  | RF | 591 | 91 | 637 | 56 |
| Zakariah et al. | 2023 | UMAP, NN | 46 | 0 | 94 | 2 |

LR Logistic regression; RF Random forest; SVM Support vector machine; CNN Convolutional neural network; DNN Deep neural network; XGBoost Extreme gradient boosting; AdaBoost Adaptive boosting; C Tree Conditional tree; KNN K-nearest neighbor; Alex Net Alex krizhevsky’s network; VGG16 Visual geometry group 16-layer; ResNet50 Residual network with 50 Layers; CNN-LSTM Convolutional neural network - Long short-term memory; CNN-GRU Convolutional neural network-gated recurrent unit; UMAP Uniform manifold approximation and projection; NN Neural network; TP true positive; TN true negative; FP false positive; FN false positive;

Figure S1: Radar chart of algorithms used in included studies. RF, random forest; NB, Naive Bayes; LR, Logistic Regression; KNN, K-nearest neighbor; CNN, Convolutional Neural network;DNN, Deep Neural Network; AdaBoost Adaptive, boosting; VGG16, Visual geometry group 16-layer; ResNet50, Residual network with 50 Layers; CNN-LSTM, Convolutional neural network - Long short-term memory; CNN-GRU, Convolutional neural network - gated recurrent unit; CNN, Convolutional neural network; Alex Net Alex, krizhevsky’s network; C Tree, Conditional tree; XGBoost, Extreme gradient boosting; UMAP, Uniform manifold approximation and projection; NN, Neural network; SVM, Support vector machine.


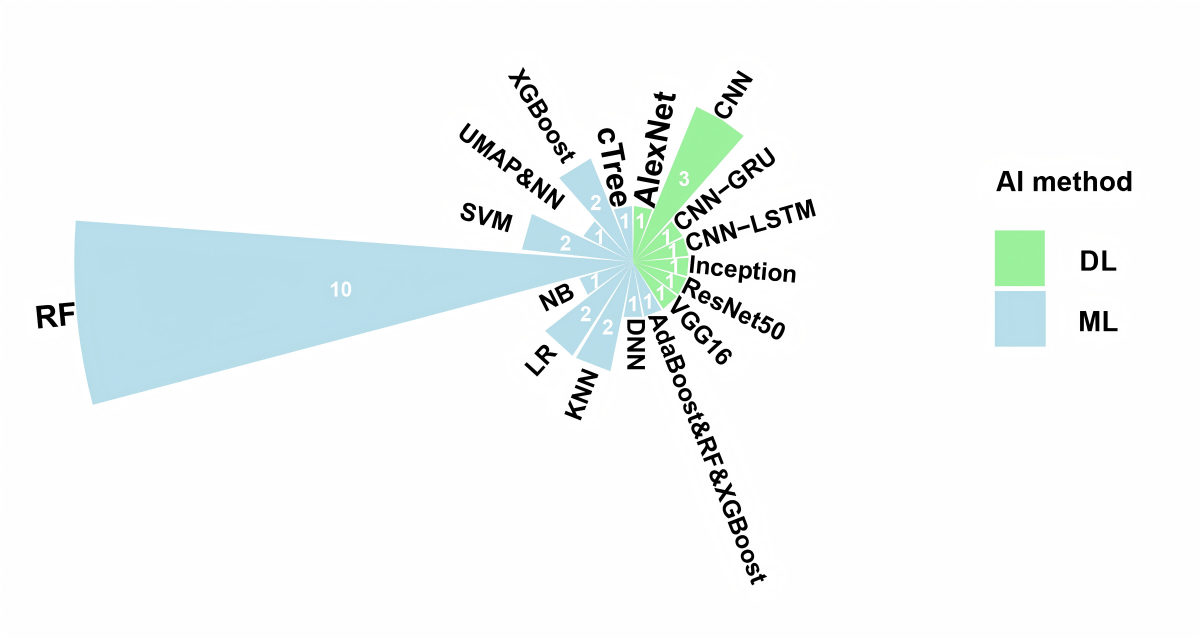


Figure S2: Bubble chart of changes in diagnostic odds ratio (DOR) over time for different algorithms. The x-axis shows the publication year and the y-axis indicates the DOR. Different colors represent different algorithms. Alex Net Alex, krizhevsky’s network; NB, Naive Bayes; CNN, Convolutional neural network; ResNet50, Residual network with 50 Layers; C Tree, Conditional tree; RF, random forest; DNN, Deep neural network; SVM, Support vector machine; UMAP, Uniform manifold approximation and projection; NN, Neural network; KNN, K-nearest neighbor; VGG16, Visual geometry group 16-layer; LR, Logistic regression; XGBoost, Extreme gradient boosting.


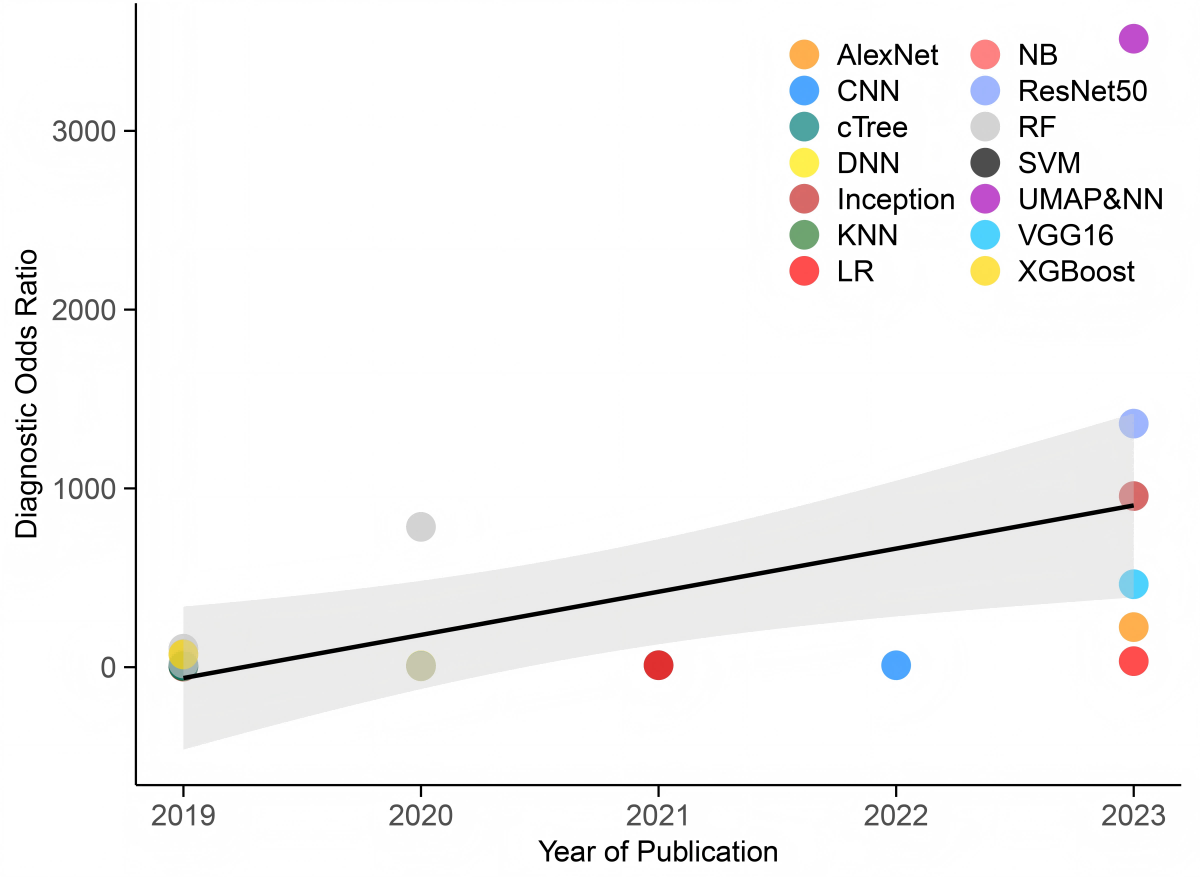


Table S7: Meta-analysis based on different AI algorithms.

| AI algorithms | Studies, n | Sensitivity(95%CI) | Specificity(95%CI) | AUC (95%CI) |
| --- | --- | --- | --- | --- |
| LR | 2 | 0.84(0.56, 0.95) | 0.80(0.41, 0.96) | NA |
| RF | 9 | 0.89(0.81, 0.94) | 0.91(0.80, 0.96) | 0.97(0.95, 0.98) |
| SVM | 2 | 0.63(0.31, 0.87) | 0.81(0.42, 0.96) | NA |
| CNN | 3 | 0.86(0.65, 0.95) | 0.91(0.69, 0.98) | NA |
| XG Boost | 2 | 0.80(0.52, 0.94) | 0.87(0.55, 0.97) | NA |
| DNN | 1 | 0.52(0.31, 0.73) | 0.81(0.64, 0.93) | NA |
| KNN | 1 | 0.74(0.52, 0.90) | 0.66(0.47, 0.81) | NA |
| NB | 1 | 0.74(0.52, 0.90) | 0.47(0.29, 0.65) | NA |
| VGG16 | 1 | 0.94(0.80, 0.99) | 0.97(0.83, 1.00) | NA |
| Alex Net | 1 | 0.94(0.85, 0.98) | 0.93(0.78, 0.99) | NA |
| Inception | 1 | 0.97(0.85, 1.00) | 0.97(0.85, 1.00) | NA |
| ResNet50 | 1 | 0.97(0.85, 1.00) | 1.00(0.88, 1.00) | NA |
| CNN-LSTM | 1 | 1.00(0.90, 1.00) | 1.00(0.88, 1.00) | NA |
| CNN-GRU | 1 | 1.00(0.90, 1.00) | 1.00(0.88, 1.00) | NA |
| UMAP&NN | 1 | 0.96(0.86, 0.99) | 1.00(0.96, 1.00) | NA |

AUC area under curve; NA not available.LR Logistic regression; LR Logistic regression; RF Random forest; SVM Support vector machine; CNN Convolutional neural network; DNN Deep neural network; XGBoost Extreme gradient boosting; AdaBoost Adaptive boosting; C Tree Conditional tree; KNN K-nearest neighbor; Alex Net Alex krizhevsky’s network; VGG16 Visual geometry group 16-layer; ResNet50 Residual network with 50 Layers; CNN-LSTM Convolutional neural network - Long short-term memory; CNN-GRU Convolutional neural network - gated recurrent unit; UMAP Uniform manifold approximation and projection; NN Neural network.

Figure S3: Fagan plot of diagnostic performance of wearable device-based AI models for depression in the internal validation set.


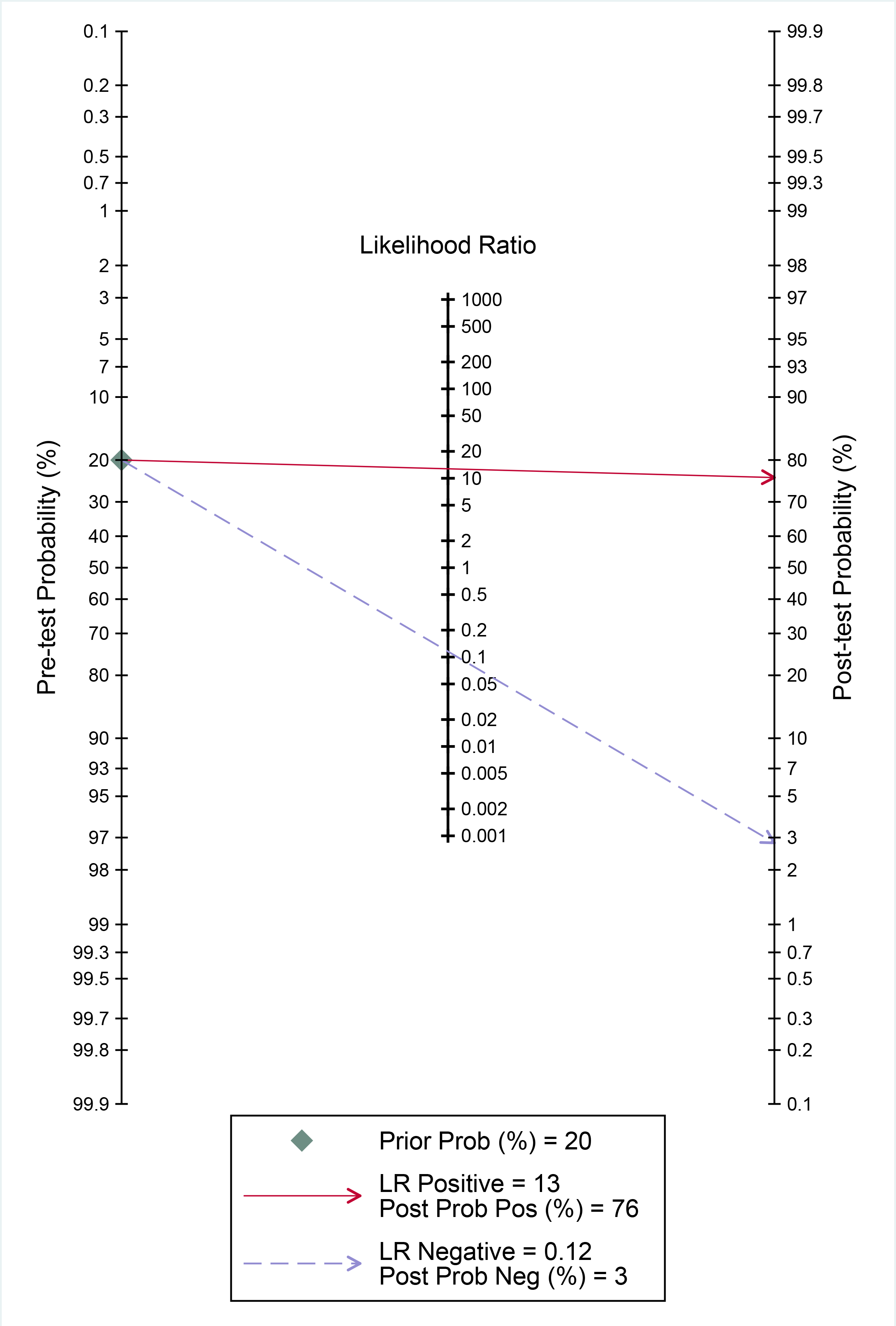


Figure S4: Deeks’ funnel plot assessing publication bias for the diagnostic performance of wearable device-based AI models for depression in the internal validation set.


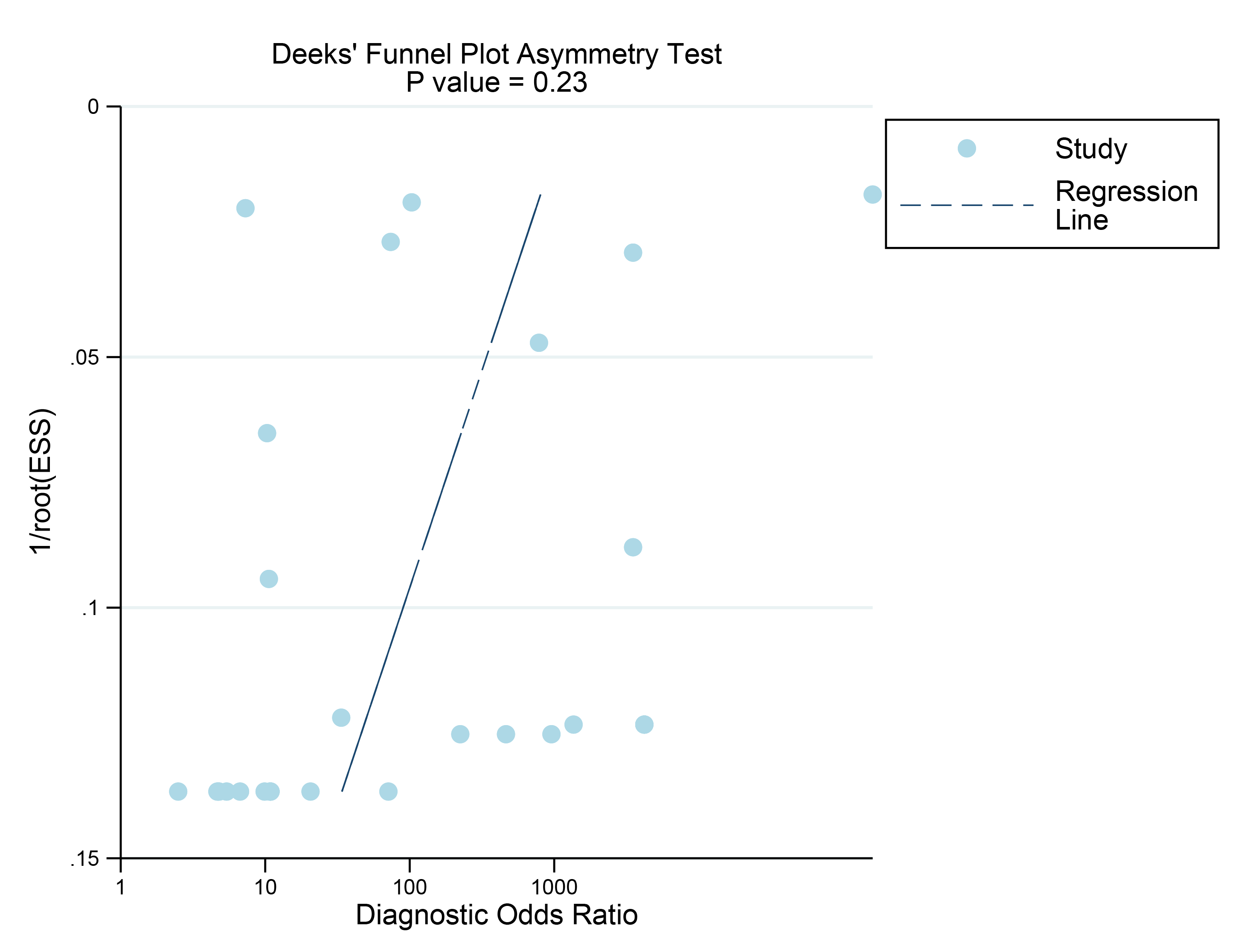


Table S8: Subgroup analysis and meta-regression analysis of wearable device-based artificial intelligence performance for detecting depression.

| **Subgroup** | **Studies, n** | **Sensitivity(95%CI****)** | **Subgroup difference** | **Specificity(95%CI)** | **Subgroup difference** |
| --- | --- | --- | --- | --- | --- |
| **Study design** |  |  | Z = 2.91, *P* = .003 |  | Z = 2.33, *P* = .01 |
| Retrospective | 19 | 0.83 (0.75,0.91) |  | 0.88(0.80,0.96) |  |
| Prospective | 10 | 0.96(0.93,1.00) |  | 0.98(0.95,1.00) |  |
| **Reference standard** |  |  | Z = 2.91, *P* = .003 |  | Z = 2.33, *P* = .01 |
| MADRS | 19 | 0.83 (0.75,0.91) |  | 0.88(0.80,0.96) |  |
| Other reference standards | 10 | 0.96(0.93,1.00) |  | 0.98(0.95,1.00) |  |
| **AI method** |  |  | Z = 2.67, *P* = .007 |  | Z = 2.37, *P* = .01 |
| Machine learning | 20 | 0.85(0.78,0.92) |  | 0.89(0.82,0.96) |  |
| Deep learning | 9 | 0.96(0.92,1.00) |  | 0.98(0.95,1.00) |  |
| **Data input** |  |  | Z = 2.91, *P* = .003 |  | Z = 2.33, *P* = .01 |
| Only activity data | 19 | 0.83(0.75,0.91) |  | 0.88(0.80,0.96) |  |
| Others | 10 | 0.96(0.93,1.00) |  | 0.98(0.95,1.00) |  |
| **Data-set source** |  |  | Z = 0.12, *P* = .89 |  | Z = 0.27, *P* = .78 |
| Open | 26 | 0.89(0.84,0.95) |  | 0.93(0.89,0.98) |  |
| Closed | 3 | 0.88(0.71,1.00) |  | 0.91(0.73,1.00) |  |

AI artificial intelligence; MADRS the Montgomery–Åsberg Depression Rating Scale.

Figure S5: Forest plot of subgroup analysis for depressive episodes.


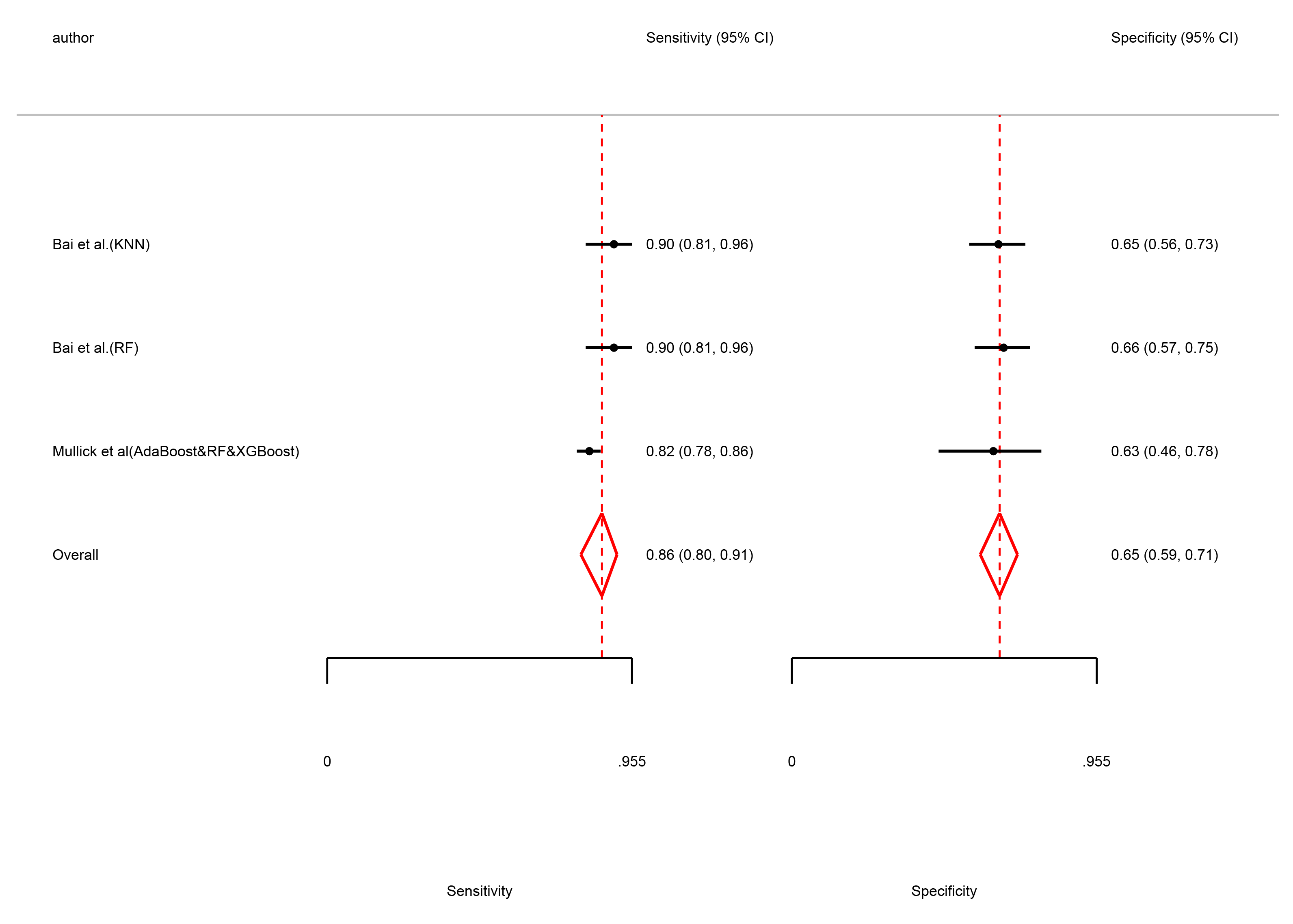

Supplement: Multimedia Appendix 1 [file mental-v13-e85319-s001.docx]
